# Exercise-Associated Gut Microbiota Remodeling and Cardiovascular Protection: Potential Mechanisms Involving Inflammatory Regulation

**DOI:** 10.3390/metabo16100766

**Published:** 2026-10-12

**Authors:** Xinxin Liu, Dandan Jing, Xuan Liu, Shujuan Hu, Xianwang Wang

**Affiliations:** 1 School of Education and Physical Education, Yangtze University, Jingzhou 434023, China; 2025720204@yangtzeu.edu.cn (X.L.); 2025720190@yangtzeu.edu.cn (D.J.); 2 School of Education, Jingzhou University, Jingzhou 434020, China; 011814@jzun.edu.cn; 3 Department of Biochemistry and Molecular Biology, Center for Molecular Medicine, Health Science Center, Yangtze University, Jingzhou 434023, China

**Keywords:** gut microbiota, microbial metabolites, cardiovascular disease, inflammation, exercise

## Abstract

Cardiovascular disease (CVD) remains a major contributor to global mortality and disease burden and encompasses a range of conditions commonly associated with metabolic disturbances, endothelial dysfunction, and chronic inflammation. Emerging evidence suggests that persistent low-grade inflammation contributes to the development and progression of CVD and represents an important component of its pathophysiology. The gut microbiota (GM) and microbiota-related metabolites are increasingly recognized as important regulators of host metabolism and immune homeostasis. GM dysbiosis may influence cardiovascular inflammation through alterations in microbial metabolites, intestinal barrier function, and inflammatory signaling, including the TLR4/NF-κB/NLRP3 pathway and immunoregulatory mechanisms involving IL-10 and context-dependent TGF-β signaling. Accumulating experimental and clinical evidence suggests that exercise may reshape GM composition and microbial metabolic profiles and may be associated with changes in inflammatory and immunoregulatory responses relevant to cardiovascular health. Evidence from atherosclerosis, hypertension, myocardial infarction, and myocardial ischemia–reperfusion injury further supports disease-specific associations among exercise, GM alterations, inflammatory signaling, and cardiovascular outcomes. Therefore, this review synthesizes current mechanistic, animal, and human evidence on these interconnected processes, with particular emphasis on the TLR4/NF-κB/NLRP3 and IL-10/TGF-β-related signaling pathways. Overall, current evidence supports disease-specific associations and potential mechanistic links among exercise, GM alterations, microbial metabolites, inflammatory regulation, and cardiovascular outcomes; however, the GM has not yet been established as a universal or causal mediator of exercise-related cardiovascular protection.

## 1. Introduction

Cardiovascular disease (CVD) remains a leading cause of global mortality and disease burden, representing a significant challenge to public health worldwide. Atherosclerosis (AS), hypertension, diabetes mellitus (DM), dyslipidemia, and chronic inflammation are closely associated with the development and progression of CVD [1].

According to the Report on Cardiovascular Health and Diseases in China 2025, CVD remained the leading cause of death among both rural and urban residents in China, accounting for 48.98% and 47.35% of all deaths in these populations, respectively, in 2021 [2]. Increasing evidence suggests that chronic low-grade inflammation is one of the key pathological mechanisms underlying the initiation and progression of CVD. The development of CVD involves multiple pathological processes, including oxidative stress, inflammation, apoptosis, autophagy, and endothelial dysfunction [3]. Inflammation contributes to the initiation and progression of AS, including plaque formation, destabilization, and thrombotic complications [4]. The gut microbiota (GM) is increasingly recognized as an important regulatory hub linking metabolic dysfunction and inflammatory responses [5]. GM dysbiosis has been associated with impaired intestinal barrier integrity and increased translocation of lipopolysaccharide (LPS) into the circulation, which may contribute to metabolic endotoxemia and systemic inflammation [6]. Meanwhile, reduced levels of beneficial metabolites, such as short-chain fatty acids (SCFAs), together with dysregulation of the GM-dependent trimethylamine (TMA)/trimethylamine N-oxide (TMAO) pathway, may further aggravate inflammation and endothelial dysfunction, thereby contributing to CVD progression [7]. Thus, modulation of GM composition and microbiota-related metabolites has attracted increasing attention as a potential strategy for CVD prevention and management.

Exercise is a safe, economical, and widely recommended non-pharmacological intervention that can attenuate pathological cardiovascular remodeling and improve cardiovascular function in experimental and clinical settings [8]. Regular exercise has been associated with alterations in GM composition, increased SCFA production, modulation of bile acid (BA) metabolism, and reduced LPS levels, changes that may contribute to reduced systemic inflammation [9]. In addition, exercise decreases the expression of tumor necrosis factor-α (TNF-α) and interleukin-6 (IL-6) in vascular smooth muscle cells (VSMCs), restores intestinal barrier integrity, and maintains intestinal homeostasis [10]. Although previous reviews have separately summarized the associations among exercise, GM, microbial metabolites, and cardiovascular health [11,12], the relationships among exercise-associated GM alterations, microbiota-related metabolites, inflammatory signaling, and cardiovascular outcomes remain incompletely characterized across different cardiovascular conditions. In particular, evidence concerning pro-inflammatory pathways, such as the Toll-like receptor 4 (TLR4)/NF-κB/NOD-like receptor protein 3 (NLRP3) axis and immunoregulatory mechanisms involving IL-10 and context-dependent transforming growth factor-β (TGF-β) signaling has not been systematically evaluated in relation to exercise-associated cardiovascular effects. Accordingly, this review focuses on the potential role of exercise-associated GM remodeling in cardiovascular inflammatory regulation, with particular emphasis on AS, hypertension, myocardial infarction (MI), and myocardial ischemia-reperfusion injury (MIRI). We synthesize evidence from human and animal studies involving different exercise modalities, including aerobic, resistance, and interval exercise, and examine key microbiota-related metabolites, particularly SCFAs, TMA/TMAO, and BAs. By synthesizing evidence on microbial composition, microbiota-related metabolites, and inflammatory and immunoregulatory signaling, this review aims to clarify the potential contribution of exercise-associated GM changes to cardiovascular protection while distinguishing disease-specific findings and current evidence limitations.

## 2. Methods

This article was conducted as a narrative review rather than a systematic review. To provide a broad and up-to-date overview of the available evidence, relevant literature was searched in PubMed, Web of Science Core Collection, and China National Knowledge Infrastructure (CNKI) from database inception to August 2026. Particular attention was given to studies published within the past five years, while earlier studies providing important foundational or mechanistic evidence were also considered. Search terms included combinations of “gut microbiota,” “exercise,” “cardiovascular disease,” “atherosclerosis,” “hypertension,” “myocardial infarction,” “myocardial ischemia–reperfusion injury,” “inflammation,” “short-chain fatty acids,” “trimethylamine N-oxide,” “bile acids,” “Toll-like receptor 4,” “nuclear factor kappa B,” “NOD-like receptor protein 3,” “interleukin-10,” and “transforming growth factor-β.” Corresponding Chinese terms were used for the CNKI search. Relevant human studies, animal studies, and mechanistically relevant in vitro studies addressing the relationships among exercise, GM and its metabolites, inflammatory regulation, and cardiovascular outcomes were considered. Publications in English or Chinese were included when they were relevant to the scope of this review. The selected literature was narratively synthesized according to the type of evidence (human, animal, or in vitro), cardiovascular condition, exercise intervention, GM alterations, microbial metabolites, and inflammation-related mechanisms. Given the narrative nature of this review, no formal systematic-review screening flow, quantitative study-selection process, or standardized quality or risk-of-bias assessment was performed.

## 3. Gut Microbiota

### 3.1. Composition and Physiological Functions of the Gut Microbiota

The GM is a complex microbial community that colonizes the human gastrointestinal tract and is primarily composed of bacteria, fungi, and viruses [13]. In healthy adults, the GM is dominated by the phyla *Firmicutes*, *Bacteroidetes*, *Actinobacteria*, and *Proteobacteria*, which collectively maintain intestinal microbial homeostasis and participate in a variety of physiological activities [14,15]. GM can metabolize dietary precursors, including choline and L-carnitine, to generate TMA [16]. Following intestinal absorption, TMA is oxidized predominantly by hepatic flavin-containing monooxygenase 3 (FMO3) to form TMAO [17]. The main byproducts of microbial carbohydrate fermentation are SCFAs, primarily acetate, propionate, and butyrate, with acetate being the most prevalent. By activating the G protein-coupled receptors GPR41 and GPR43, SCFAs may contribute to improved intestinal barrier integrity, reduced intestinal inflammation, and regulation of inflammatory responses and glucose–lipid metabolism. Primary BAs are synthesized from cholesterol in the liver and subsequently undergo microbial transformations in the intestine, including deconjugation and 7α-dehydroxylation, which contribute to the formation of the secondary BA pool. BAs also act as signaling molecules through the nuclear receptor farnesoid X receptor (FXR) and the membrane receptor TGR5, thereby influencing host metabolic regulation [18]. In addition to microbial metabolites, specific beneficial bacteria can directly contribute to intestinal homeostasis. For instance, by creating a biological barrier, preventing pathogen adhesion and proliferation, and lowering intestinal permeability, the promising next-generation probiotic *Akkermansia muciniphila* improves intestinal barrier function. SCFAs also contribute to metabolic homeostasis by providing energy to intestinal epithelial cells, participating in lipid and cholesterol metabolism, and regulating insulin sensitivity [19]. Additionally, GM may contribute to the regulation of excessive inflammatory responses and the maintenance of immunological homeostasis through effects on immune cell differentiation and the development of gut-associated lymphoid tissue [20]. Overall, GM dysbiosis may promote inflammation and disrupt host homeostasis.

### 3.2. Gut Microbiota and Inflammation

Changes in the composition and functional status of GM, a crucial regulator of the host immune system, are closely linked to inflammatory responses [21]. GM dysbiosis modulates systemic inflammatory responses through various signaling pathways in addition to causing local intestinal inflammation. Available evidence suggests that GM dysbiosis may be associated with increased LPS production and enhanced translocation of LPS into the systemic circulation through a compromised gut barrier. Following LPS binding to TLR4, which is expressed on intestinal and immune cells, the adaptor protein myeloid differentiation primary response protein 88 (MyD88) is recruited to activate downstream inflammatory signaling. This activation of the nuclear factor kappa B (NF-κB) signaling pathway results in the production of pro-inflammatory cytokines, such as TNF-α, interleukin-1β (IL-1β), and IL-6 [22,23,24]. Furthermore, oxidized low-density lipoprotein (ox-LDL) accelerates atherosclerotic plaque development and inflammation by activating the NLRP3 inflammasome and downstream caspase-1, thereby promoting interleukin-1β (IL-1β) maturation and TNF-α release [24,25]. At the microbial level, a higher abundance of *Tyzzerella* has also been associated with an increased risk of CVD [26]. LPS administration has been shown in animal studies to cause intestinal mucosal villus edema, inflammatory cell infiltration within the lamina propria, and decreased expression of zonula occludens-1 (ZO-1), along with increased expression of inflammatory genes, such as IL-1β and interleukin-8 (IL-8) [23].

In contrast, beneficial microbial metabolites, particularly SCFAs, may contribute to immune homeostasis and attenuation of inflammatory responses. Exogenous acetate, propionate, and butyrate have been shown to suppress NLRP3/Caspase-1 signaling, reduce inflammatory mediator production, alleviate cellular inflammatory injury, and improve metabolic function in Ox-LDL-stimulated THP-1 cells [27]. Sun et al. found that high-molecular-weight heteropolysaccharides altered GM composition, triggered PTEN-induced kinase 1 (PINK1)/Parkin RBR E3 ubiquitin protein ligase (PRKN)-dependent mitophagy, blocked NLRP3 inflammasome activation, and reduced inflammation in a mouse model of acute colitis [28]. Moreover, in an *Escherichia coli*-induced colitis mouse model, combined treatment with *Lactobacillus amylovorus* and baicalin increased colonic *Lactobacillus* abundance and levels of indole-related metabolites, inhibited TLR4/STAT signaling, and reduced M1 macrophage polarization [29]. Clinical investigations have also reported that greater abundance of SCFA-producing bacteria is associated with higher interleukin-10 (IL-10) expression, lower prevalence of potentially harmful bacteria such as *Escherichia coli* and *Shigella*, and a more favorable inflammatory profile in patients with ulcerative colitis (UC) [30]. Changes in inflammatory biomarkers further support an association between GM dysbiosis and systemic inflammation. Elevated C-reactive protein (CRP) levels, a well-known measure of systemic inflammation, are usually associated with decreased GM diversity and altered microbial composition [31]. In a mouse model of colitis, elevated levels of CRP, IL-1β, and IL-6 were accompanied by significant alterations in the abundance of several bacterial taxa, including *Bacteroides*, *Clostridium*, *Enterobacteriaceae*, and related microbial communities [32]. Moreover, obese children exhibited significantly elevated CRP levels together with reduced gut microbial diversity [33]. Collectively, these findings support a close association between GM dysbiosis and local and systemic inflammatory responses.

## 4. Roles of the GM in CVD

Accumulating evidence suggests that GM dysbiosis is associated with the development and progression of CVD. Alterations in GM composition and metabolic activity have been associated with disturbances in lipid metabolism, inflammatory responses, and vascular endothelial homeostasis, which may contribute to CVD progression [34]. CVD encompasses diverse disorders, including AS, hypertension, and MI. AS is characterized by endothelial dysfunction, lipid accumulation, plaque formation, and chronic vascular inflammation, resulting in arterial stiffening and luminal narrowing. Hypertension, a major cardiovascular risk factor, impairs endothelial function and accelerates the development of coronary artery and cerebrovascular diseases. MI, a severe manifestation of coronary artery disease (CAD), often involves myocardial ischemia–reperfusion injury (MIRI), in which the restoration of blood flow paradoxically aggravates myocardial damage [35,36]. Beyond changes in microbial composition, GM-derived bioactive metabolites have been implicated in CVD progression through specific receptor-mediated pathways [37].

### 4.1. Gut Microbiota and AS

AS is characterized by vascular endothelial injury, lipid deposition, and chronic inflammation. Accumulating evidence suggests that GM dysbiosis is associated with the development and progression of AS. Jie et al. included 218 patients with atherosclerotic CVD and 187 healthy controls in their study. They found that the number of *Enterobacteriaceae* increased, while the number of butyrate-producing bacteria such as *Roseburia intestinalis* decreased [38]. A culture-based study in elderly patients with CAD also reported higher counts of cultivable *Enterobacteriaceae* and *Enterococcus* and lower counts of *Bifidobacterium*, *Lactobacillus*, and *Bacteroides* than in healthy controls [39]. These findings indicate associations rather than causal effects of individual taxa. Animal studies have further shown that co-administration of *Lactobacillus rhamnosus* and *Lactobacillus acidophilus* via gavage can reduce atherosclerotic plaque formation in mice [40]. Similarly, supplementation with *Pediococcus pentosaceus* in high-fat-diet-fed mice reduced aortic plaque area, decreased serum TNF-α and IL-6 levels, increased IL-10 expression, and markedly elevated the production of indole-3-pyruvic acid (I3P) [41,42,43].

In addition to changes in bacterial community composition, alterations in microbiota-related metabolites may also contribute to inflammatory responses. Decreased levels of SCFAs, together with alterations in the GM-dependent TMA/TMAO pathway and increased levels of potentially harmful metabolites such as phenylacetylglutamine (PAGln) and imidazole propionate (ImP), may contribute to a pro-inflammatory and pro-thrombotic metabolic environment. Among these metabolites, PAGln can enhance platelet reactivity and has been associated with an increased risk of cardiovascular events [44,45,46,47]. SCFAs may contribute to vascular endothelial homeostasis and lipid metabolism, whereas TMAO has been implicated in foam cell formation and reduced plaque stability [48,49,50,51]. In addition, FXR is involved in the regulation of AS, and recent evidence suggests that microbiome-dependent alterations in BA metabolism may influence AS through FXR-dependent signaling [52]. The dual FXR/TGR5 agonist INT-767 has been shown to attenuate atherosclerotic lesion formation and inflammatory responses; mechanistically, TGR5 activation enhances cAMP/PKA signaling, thereby suppressing NF-κB activation and reducing pro-inflammatory cytokine production [53]. However, other studies have reported that FXR deficiency also protects mice against AS development. These apparently divergent findings suggest that the role of FXR in AS may be tissue- and context-dependent, and the underlying regulatory mechanisms require further investigation [54].

### 4.2. Gut Microbiota and Hypertension

Hypertension is a chronic disease characterized by elevated systemic arterial blood pressure and represents an important risk factor for the incidence of CVD and premature mortality. Multiple factors, including genetic and environmental factors, influence its development [55]. Recent studies have shown that GM imbalance is closely associated with abnormal blood pressure and may contribute to the development of hypertension by modulating inflammatory responses, metabolic functions, and vascular homeostasis. Therefore, modulation of GM has attracted increasing interest as a potential approach for hypertension prevention and management [56].

Human observational studies have reported reduced microbial richness and diversity and altered GM composition in individuals with hypertension [57]. However, these findings should not be interpreted as a uniform microbial signature of hypertension. Changes in SCFA concentrations also require distinction between biological compartments. A cross-sectional study reported lower concentrations of several plasma SCFAs but higher fecal SCFA concentrations in hypertensive participants than in normotensive controls [58]. SCFAs may exert protective effects by supporting endothelial homeostasis, promoting vascular relaxation, and modulating inflammatory responses. Beyond their vascular protective and anti-inflammatory effects, SCFAs may also regulate blood pressure through neurohumoral pathways. SCFA-mediated activation of GPR41 (FFAR3) promotes vasodilation, whereas Olfr78 activation stimulates renin release, indicating receptor-dependent regulation of blood pressure [59,60]. In spontaneously hypertensive rats, acetate supplementation attenuated neuroinflammation in the rostral ventrolateral medulla (RVLM), reduced sympathetic activity, and lowered arterial blood pressure, suggesting that central sympathetic regulation may represent another pathway linking SCFAs to blood pressure control [61]. However, human intervention studies have yielded contrasting results. In a randomized crossover trial involving untreated patients with essential hypertension, three weeks of supplementation with acetylated and butyrylated high-amylose maize starch reduced 24-h systolic blood pressure (SBP) and increased circulating acetate and butyrate levels [62]. By contrast, a separate randomized trial found that four weeks of oral sodium butyrate supplementation increased daytime systolic and diastolic blood pressure (DBE) compared with placebo [63]. In contrast, TMAO, a detrimental GM-dependent metabolite generated through microbial TMA production and subsequent hepatic oxidation, has been implicated in vascular inflammation, enhanced Ang II-induced hypertensive responses, platelet activation, and vascular dysfunction [64,65]. Clinical investigations have revealed that elevated circulating TMAO levels are positively associated with hypertension risk. Nevertheless, reductions in circulating TMAO levels may be associated with improved blood pressure control and decreased cardiovascular event risk [66]. The balance between protective metabolites such as SCFAs and detrimental metabolites such as TMAO may influence the regulatory effects of GM on blood pressure homeostasis [67].

### 4.3. Gut Microbiota and MI

MI is an acute cardiovascular event characterized by acute myocardial injury with clinical evidence of myocardial ischemia, typically resulting from acute coronary artery occlusion and leading to myocardial necrosis and subsequent inflammatory responses [68]. Type 1 MI is commonly associated with acute atherothrombotic coronary obstruction following the disruption of an AS plaque. Recent studies have found that significant alterations in intestinal microbiota composition in MI patients are associated with disease progression and may predict subsequent CVD events. The specific changes are shown in Table 1 [69].

Animal studies have further demonstrated that probiotic intervention restores *Firmicutes* abundance in MI mice, reduces the relative proportions of *Bacteroidetes*, *Verrucomicrobia*, and *Actinobacteria*, and promotes the recovery of GM homeostasis [70]. Su et al. reported that *Morchella* polysaccharide treatment enriched intestinal *Lactobacillus*, increased 12-hydroxyeicosapentaenoic acid (12-HEPE) levels, and attenuated post-MI inflammation in mice. Antibiotic-mediated microbiota depletion abolished the treatment-induced increase in 12-HEPE and its anti-inflammatory effects, supporting microbiota involvement in this response [71] (Table 1). Elevated circulating TMAO levels, generated through the GM-dependent TMA/TMAO metabolic pathway, are associated with adverse cardiovascular outcomes after MI. Experimental studies suggest that inhibition of microbial TMAO production or reduction of circulating TMAO levels may attenuate adverse cardiac remodeling and improve cardiac function after cardiac injury [72]. Li et al. further showed that reducing circulating TMAO levels delayed MI-induced heart failure progression in rats, which was accompanied by decreased IL-8 expression and secretion, suggesting that TMAO may contribute to post-MI inflammatory responses [73]. Meanwhile, bacterial translocation, intestinal permeability, systemic inflammation, and myocardial injury were all alleviated in mice [44]. Clinical studies further support the protective role of metabolites in MI. Oral administration of lactulose solution to AMI patients can promote the generation of SCFAs and reduce systemic inflammation, thereby restoring cardiac function [74]. In summary, GM dysbiosis and metabolite imbalance may contribute to MI progression. SCFAs and TMAO may represent potential targets for promoting myocardial repair and preventing chronic heart failure after MI.

### 4.4. Gut Microbiota and Myocardial Ischemia–Reperfusion Injury

MIRI is a common and severe complication following reperfusion therapy for AMI. It is characterized by progressive inflammatory injury and deterioration of cardiac function despite the restoration of coronary blood flow [75]. In addition to local myocardial inflammation, it is also closely associated with GM dysbiosis.

In a mouse model of MIRI, alterations in GM composition included an increased relative abundance of *Escherichia* and a decreased relative abundance of *Lactobacillus* [76]. GM dysbiosis may be associated with increased release of microbial metabolites and bacterial components, which may contribute to myocardial injury. Bacterial extracellular vesicles (EVs) are nanoscale membrane vesicles released by bacteria that can transport bioactive cargo, including LPS, and serve as important mediators of GM–host communication [77]. Under dysbiotic conditions, alterations in the intestinal microenvironment and increased microbial stress may favor bacterial EV release, thereby facilitating the delivery of bacterial components such as LPS to the host. In particular, EVs derived from *Escherichia coli* can carry LPS into the systemic circulation and may promote the recruitment of systemic and local inflammatory cells, thereby contributing to myocardial inflammation [78]. Furthermore, dipeptidyl peptidase-4 (DPP-4) produced by *Bacteroides acidifaciens* may promote glucagon-like peptide-1 (GLP-1) degradation and thereby reduce its cardioprotective effects, whereas DPP-4 inhibition may facilitate cardiac functional recovery [79]. Flavonoids derived from commensal bacteria and their metabolite desaminotyrosine suppress the pro-inflammatory activity of macrophages by reducing IL-6 production [80]. Wang et al. reported that Xintong Granules were associated with modulation of GM and increased intestinal SCFA levels, including acetate and propionate, in rats with MIRI. These changes were accompanied by reduced myocardial infarct size, lower CK-MB and CK levels, and decreased inflammatory cytokine expression [81]. Animal studies have further suggested that *Lactobacillus* and several of its strains may be associated with reduced CRP levels, attenuated myocardial inflammation, smaller infarct size, and improved cardiac function in MIRI [42,82]. In addition, *Limosilactobacillus reuteri* and gamma-aminobutyric acid attenuate cardiac inflammation by inhibiting lysosomal leakage and NLRP3 inflammasome activation, thereby reducing M1 macrophage polarization [83].

## 5. Exercise and Gut Microbiota

Epidemiological, clinical, and animal studies have shown a bidirectional regulatory relationship between exercise and GM [84]. Regular exercise not only improves cardiovascular metabolic indicators, inhibits inflammatory responses and enhances insulin sensitivity but also reduce the risk of CVD [85].

Exercise has been associated with alterations in GM composition, including enrichment of potentially beneficial taxa such as *Bifidobacterium*, *Lactobacillus*, and *Faecalibacterium*, as well as changes in potentially pathogenic taxa such as *Streptococcus* and *Klebsiella* [86]. In this context, treadmill exercise preconditioning restored GM diversity, increased the abundance of beneficial bacteria such as *Lactobacillus*, and attenuated intestinal barrier disruption in rats with ischemic stroke [87,88]. Aerobic exercise, resistance exercise, and combined exercise increased microbial diversity, enriched SCFA-producing bacteria, and reduced LPS levels in mice with T2DM [89]. These changes were accompanied by improvements in metabolic parameters [90]. Moreover, exercise intervention has been shown to remodel GM composition in CVD models. In mice with MI, moderate-intensity exercise altered gut microbial community structure, increased beneficial taxa including *Akkermansia*, and was associated with improved cardiac function [91]. In AS-prone mice, treadmill exercise remodeled the GM, increased SCFA-producing bacterial taxa, reduced vascular inflammatory markers, and attenuated early atherosclerotic lesion development [92].

Human studies have provided complementary evidence, although GM responses vary across populations and exercise interventions. Moderate-to-high physical activity was associated with higher abundance of *Prevotella copri* [93]. Exercise combined with dietary intervention improved the gut microbial composition in children with obesity and reduced the abundance of pathogenic bacteria as well as the levels of inflammatory cytokines, including TNF-α, IL-6, and CRP [94]. In humans, structured exercise interventions have been shown to alter GM composition and functional capacity, with exercise-induced microbial changes differing according to baseline metabolic status and training characteristics [95]. Compared with sedentary patients with T2DM, physically active patients exhibited differences in GM composition, including a higher abundance of *Bacteroidetes*, a lower F/B ratio, and reduced abundances of *Clostridium* and *Blautia*, which were associated with a more favorable intestinal profile [96]. With respect to microbial metabolism, *Prevotella* was enriched in cyclists and was accompanied by enhanced carbohydrate metabolism and SCFA metabolism [97]. In patients with heart failure, three months of cardiac rehabilitation was associated with improved functional capacity and alterations in GM composition. Patients showing clinically meaningful improvements in 6-min walk distance exhibited increased microbial α-diversity and enrichment of taxa, including *Lachnospiraceae* UCG-004, *Lawsonella*, and *Ruminococcus* gnavus groups [98]. This indicates that maintaining moderate exercise, whether it is high-intensity interval training (HIIT) or moderate-intensity interval training (MICT), may help improve gastrointestinal homeostasis, reduce inflammatory responses, and minimize myocardial damage [99]. Among them, moderate-intensity continuous training (MICT) was associated with lower SBP and reduced NADPH oxidase 4 (NOX4) expression [100]. Aerobic exercise combined with blood flow restriction training can up-regulate vascular endothelial growth factor (VEGF) and insulin-like growth factor 1, promoting angiogenesis and metabolic regulation benefits [101]. (Table 2). Complementary evidence has also emerged from human studies. In patients with hypertension, 12 weeks of HIIT reduced blood pressure, arterial stiffness, and circulating TMAO while increasing acetate, propionate, and butyrate levels [102] (Table 2).

Collectively, current evidence supports an association between exercise and GM remodeling, including changes in microbial composition and metabolic function. These exercise-associated alterations may contribute to improved inflammatory homeostasis and cardiovascular health, although the extent to which GM mediates exercise-induced cardiovascular benefits requires further investigation.

**Table 1:** Relationship between gut microbiota and CVD.

Research Subject	Intervention Method	Changes in Gut Microbiota	Effect on CVD	Reference
Patients with valvular calcification (VC) and CAD	16S rRNA gene sequencing	*Veillonella dispar*, *Bacteroides plebeius*, *Fusobacterium* ↑; *Collinsella aerofaciens*, *Megamonas*, *Enterococcus* ↓	Modulating the types and quantities of GM may play a significant role in the treatment of CVD.	[103]
MI mice	Experimental animal study; *Morchella* polysaccharide administration	*Lactobacillus* ↑	GM-associated 12-HEPE ↑; post-MI cardiac inflammation ↓; cardiac repair improved	[71]
Mouse model of diabetic cardiomyopathy (DCM)	Electroacupuncture intervention	The structure of the ileum is clear, with abundant intestinal glands. The epithelial cells of the mucosa are arranged regularly, while scattered epithelial cells show damage and atrophy.	Regularly arranged cardiomyocytes, reduced inflammatory cell infiltration, and well-preserved tissue architecture.	[104]
SICM mouse model	Tonifying Qi, Activating Blood Circulation and Detoxifying Formula	*Bacillota*, *Bacteroidota ↓*, *Pseudomonadota*, *Deferribacterota ↑*	Levels of myocardial injury markers and inflammatory factors ↓	[105]
HFD + STZ-induced T2DM rats	Golden chrysanthemum tea and metformin	*Lactobacillus*, *Bacillus*, unclassified_Muribaculaceae, Prevotellaceae_UCG_001 ↑ Dubosiella, unclassified Lachnospiraceae, *Lachnospiraceae*_NK4A136_group ↓	IL-6, IL-1β, TNF-α ↓	[106]
AMI mouse model	Gavage of probiotics	*Firmicutes*, including *Lactobacillus* and *Clostridium*, ↑ *Bacteroidetes*, *Verrucomicrobia*, *Actinobacteria* ↓	IL-17, IL-23 and interferon-γ (IFN-γ) ↓	[70]
MI rat model Zadi-5	Zadi-5	*Actinomycetota*, *Clostridia* and *Bacilli* ↑ *Gammaproteobacteria*, *Acetobacter* ↓	The levels of TC, TG and low-density lipoprotein cholesterol (LDL-C) all decreased, while the level of high-density lipoprotein cholesterol (HDL-C) increased; the degree and extent of cell necrosis significantly reduced, and the number of inflammatory cells was lower.	[107]
Old C57BL/6 mice	Progressive resistance treadmill training	TMA/TMAO ↓	IFN-γ ↓, keratinocyte-derived chemokine (KC) ↓; Cardiac function recovery	[108]
SHR, Ang II-induced hypertensive rats, and patients with hypertension	SHR vs. WKY; Ang II ± minocycline	GM diversity ↓; F/B ratio ↑; acetate- and butyrate-producing bacteria ↓	GM dysbiosis was associated with hypertension; minocycline attenuated Ang II-induced hypertension	[109]

## 6. Exercise-Associated Gut Microbiota Remodeling and Inflammatory Regulation in CVD

Inflammation plays a crucial role in the pathogenesis of CVD, mainly regulating disease progression through pro-inflammatory and anti-inflammatory mechanisms. Regular exercise can systematically regulate inflammatory responses through the core regulatory axis of “exercise–GM–inflammation–cardiovascular system” [110,111,112].

### 6.1. Exercise Regulates the Gut Microbiota to Inhibit the TLR4/NF-κB/NLRP3 Pro-Inflammatory Signaling Axis and Alleviate CVD

The imbalance of the GM and its metabolites has been increasingly associated with the development and progression of CVD, although the specific microbial alterations and their clinical significance may differ across cardiovascular conditions. Human observational studies have reported reduced microbial richness and diversity, depletion of potentially beneficial bacteria such as *Lactobacillus*, *Bifidobacterium*, and *Subdoligranulum*, and enrichment of opportunistic taxa including *Tyzzerella* and *Desulfovibrio* in patients with CVD [113]. These microbial alterations have also been associated with impaired intestinal barrier function and disturbances in microbiota-related metabolites, including altered SCFA metabolism and elevated circulating TMAO levels [114]. Increased intestinal permeability may facilitate the translocation of bacterial products such as LPS into the circulation, thereby contributing to a systemic pro-inflammatory environment [115]. Mechanistic evidence further indicates that circulating LPS can activate TLR4/MyD88-dependent signaling, enhance NOX activity in vascular endothelial cells [116], induce oxidative stress, and activate the downstream IκB kinase (IKK) complex [53], thereby promoting NF-κB p65 nuclear translocation [117]. As a central transcriptional regulator of pro-inflammatory gene expression, activated NF-κB can increase the expression of inflammatory mediators and endothelial adhesion molecules, including intercellular adhesion molecule-1 (ICAM-1) and vascular cell adhesion molecule-1 (VCAM-1) [118].

**Figure 1:**
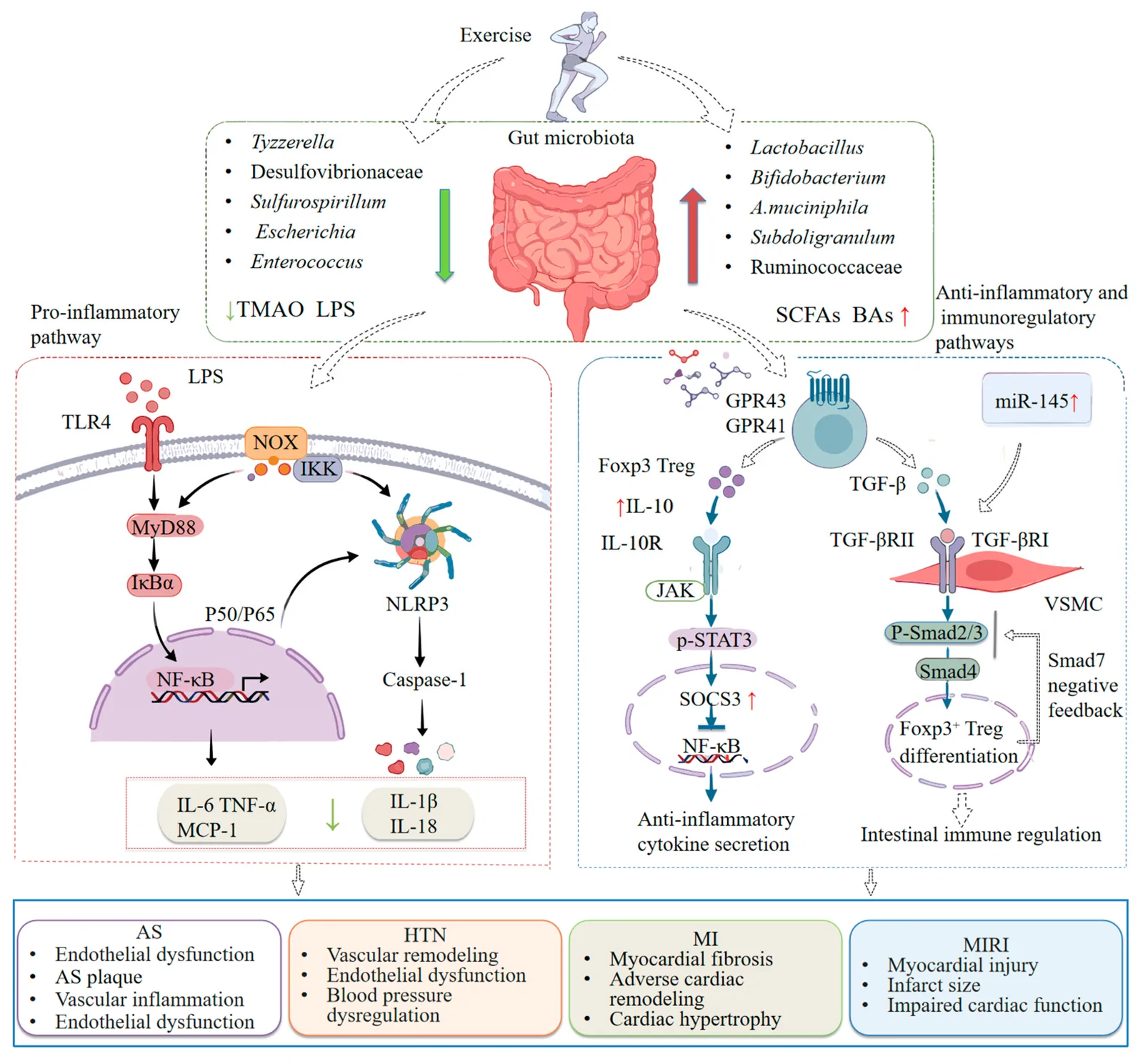
Conceptual evidence map of exercise-associated GM changes, inflammatory and immunoregulatory signaling, and disease-specific cardiovascular findings. Exercise may reshape GM composition, characterized by reduced abundances of inflammation-associated taxa, including *Tyzzerella*, *Desulfovibrionaceae*, *Sulfurospirillum*, *Escherichia*, and *Enterococcus*, together with enrichment of potentially beneficial taxa, including *Lactobacillus*, *Bifidobacterium*, *Akkermansia muciniphila*, *Subdoligranulum*, and *Ruminococcaceae*. These microbial alterations are accompanied by decreased LPS and TMAO levels and increased SCFAs and BAs. On the pro-inflammatory side, reduced LPS/TMAO-related signaling may be associated with attenuation of the TLR4/MyD88/NF-κB/NLRP3 pathway, resulting in lower expression of IL-6, TNF-α, MCP-1, IL-1β, and IL-18. On the anti-inflammatory and immunoregulatory side, SCFAs may activate GPR41/GPR43 signaling and promote Foxp3^+^ regulatory T-cell (Treg)-related responses, together with IL-10/IL-10R/JAK/STAT3/SOCS3 signaling. Exercise-associated changes may also influence context-dependent TGF-β signaling, including miR-145-related regulation, TGF-β receptor/Smad2/3/Smad4 signaling, and Smad7 negative feedback, thereby contributing to intestinal immune regulation and potentially limiting excessive profibrotic activation in VSMCs. The lower panels summarize the disease categories emphasized in this review and their most relevant associated outcomes: atherosclerosis (AS; endothelial dysfunction, plaque formation, and vascular inflammation), hypertension (HTN; vascular remodeling, endothelial dysfunction, and blood pressure dysregulation), myocardial infarction (MI; myocardial fibrosis, adverse cardiac remodeling, and cardiac hypertrophy), and myocardial ischemia–reperfusion injury (MIRI; myocardial injury, infarct size, and impaired cardiac function). Dashed arrows indicate proposed or incompletely established relationships, whereas solid arrows indicate relatively well-established molecular signaling relationships. Abbreviations: GM, gut microbiota; SCFAs, SCFA; BAs, bile acids; LPS, lipopolysaccharide; TMAO, trimethylamine N-oxide; TLR4, Toll-like receptor 4; MyD88, myeloid differentiation primary response 88; NF-κB, nuclear factor-kappa B; NLRP3, NOD-like receptor protein 3; GPR41/GPR43, G protein-coupled receptor 41/43; Treg, regulatory T cell; IL-10, interleukin-10; IL-10R, interleukin-10 receptor; JAK, Janus kinase; STAT3, signal transducer and activator of transcription 3; SOCS3, suppressor of cytokine signaling 3; TGF-β, transforming growth factor-beta; AS, atherosclerosis; HTN, hypertension; MI, myocardial infarction; MIRI, myocardial ischemia–reperfusion injury.

NF-κB is a family of transcription factors, with the p50/p65 heterodimer representing the predominant form of canonical NF-κB. Under resting conditions, NF-κB is retained in the cytoplasm through its association with inhibitory IκB proteins [119]. Upon activation of upstream inflammatory signaling, degradation of IκB permits NF-κB nuclear translocation and transcriptional regulation of inflammatory genes (Figure 1). Consistent with this role, in cultured human endothelial cells, TNF-α stimulation increased ICAM-1 expression through PI3K/NF-κB-related signaling and was accompanied by enhanced monocyte adhesion [118]. Direct in vivo evidence also supports exercise-related modulation of NF-κB in AS. In ApoE-deficient mice with AS, aerobic exercise reduced aortic NF-κB protein expression and increased adiponectin receptor 1 expression, accompanied by reduced neointimal hyperplasia and atherosclerotic plaque area [120]. Additional animal studies further linked exercise with concurrent changes in GM composition, inflammatory markers, and atherosclerotic lesion development. In the same genetic background under a Western diet, 12 weeks of endurance exercise increased SCFA-associated taxa, including *Rikenellaceae* and *Dubosiella*, and SCFA production, while reducing vascular inflammatory markers and atherosclerotic lesions [121]. Four weeks of treadmill training was accompanied by changes in GM composition and circulating adhesion molecules, together with an approximately 29% reduction in early-stage atherosclerotic lesion area [92]. These animal studies indicate that exercise-related changes in GM composition can occur in parallel with reductions in vascular inflammatory markers and atherosclerotic lesion burden. However, they do not establish that GM remodeling directly mediates the anti-inflammatory or anti-atherosclerotic effects of exercise. In patients with coronary heart disease after percutaneous coronary intervention, 12 weeks of HIIT resulted in a greater improvement in 6-min walk performance than moderate-intensity continuous training and was accompanied by alterations in GM composition, including an increased abundance of *Clostridiales*, as well as changes in circulating metabolites [122]. However, human evidence remains heterogeneous, and not all inflammatory or vascular markers respond consistently to exercise. In sedentary middle-aged and older women, 8 weeks of Flexi-Bar exercise increased the abundance of beneficial bacteria, such as *Subdoligranulum* [123]. In patients with heart failure, three months of exercise-based cardiac rehabilitation was associated with improved functional capacity and increased GM α-diversity, together with enrichment of several bacterial taxa, including *Lachnospiraceae UCG-004* [98]. By contrast, findings in healthy middle-aged adults were less consistent. A 12-month aerobic exercise intervention improved cardiorespiratory fitness and reduced hair cortisol levels, whereas circulating IL-6, ICAM-1, and pulse-wave velocity did not show significant intervention effects [124]. Taken together, these human studies indicate that responses to exercise may vary according to the study population and outcomes assessed, and they do not establish whether GM remodeling directly mediates inflammatory regulation in CVD. Outside the cardiovascular setting, a metabolic disease model provides complementary evidence that exercise may be accompanied by changes in both GM composition and NF-κB/NLRP3-related inflammatory markers. In HFD-induced NAFLD mice, eight weeks of aerobic exercise remodeled the GM, including partial normalization of *Escherichia-Shigella* and *Leuconostoc*, and reduced hepatic protein expression of NF-κB, NLRP3, IL-1β, and TNF-α [125].

NLRP3 inflammasome activation is classically described by a two-signal model. In the priming phase (signal 1), activation of the TLR4/NF-κB pathway promotes the transcriptional expression of NLRP3 and pro-IL-1β, thereby preparing the inflammasome for subsequent activation [126,127]. Following this priming step, cellular stress-related stimuli, including reactive oxygen species (ROS), can provide the activation signal (signal 2), promoting NLRP3 inflammasome assembly and caspase-1 activation [127,128]. At the metabolic level, alterations in SCFAs and TMAO have been reported in different experimental settings and may contribute to a pro-inflammatory environment. Activated caspase-1 subsequently cleaves pro-IL-1β and GSDMD, promoting IL-1β maturation and GSDMD-mediated pyroptosis. The resulting microenvironment may promote endothelial activation and monocyte recruitment, thereby linking NLRP3-related inflammation to atherosclerotic lesion development [129]. In AS, endothelial activation and monocyte recruitment promote macrophage accumulation and foam cell formation, thereby contributing to plaque progression and instability [130]. Direct experimental evidence further supports the involvement of the microbiota-related metabolite TMAO in NLRP3-mediated vascular inflammation. In ApoE-deficient mice, dietary choline supplementation increased circulating TMAO levels and was accompanied by enhanced aortic NLRP3 and caspase-1 activation, increased expression of IL-1β, ICAM-1, and MMP-9, and a greater atherosclerotic lesion burden. Consistent with the in vivo findings, TMAO treatment in HUVECs increased IL-1β, ICAM-1, and MMP-9 expression and enhanced NLRP3/caspase-1 activation, whereas pharmacological inhibition or siRNA-mediated knockdown of NLRP3 attenuated these inflammatory responses [131]. Exercise may also modulate this inflammatory process. In Western-diet-fed mice, eight weeks of voluntary wheel running prevented the elevation of circulating TMAO and was accompanied by reduced myocardial TNF-α, increased IL-10, attenuated myocardial fibrosis, and preserved cardiac function. Importantly, TMAO supplementation during exercise attenuated these anti-inflammatory and cardioprotective effects, suggesting that TMAO may contribute to exercise-related cardiac protection [132]. Complementary evidence from a non-cardiovascular model showed that, in TMAO-treated APP/PS1 mice, 12 weeks of voluntary wheel running reduced circulating TMAO levels and improved gut microbial profiles [133].

In the context of hypertension, Ang II represents another important upstream stimulus associated with NLRP3 inflammasome activation. Ang II may promote the release of damage-associated molecular patterns (DAMPs), which can act as upstream signals for NLRP3 activation and thereby amplify vascular inflammation [134]. Animal studies have also examined exercise-associated GM and inflammatory responses in hypertensive models. In spontaneously hypertensive rats (SHRs), 12 weeks of moderate-intensity exercise reduced SBP and remodeled the GM, with enrichment of butyrate-producing taxa including *Eubacterium xylanophilum* group, *Lachnospiraceae ND3007* group, *Roseburia*, *Ruminococcaceae UCG-005*, and *RuminiClostridium 6*. These microbial changes were accompanied by improved intestinal barrier function and attenuation of intestinal and hypothalamic inflammation. Notably, fecal microbiota transplantation from exercised SHRs to sedentary SHRs also attenuated blood pressure elevation and improved the gut–brain axis, suggesting that exercise-induced GM remodeling may contribute to its antihypertensive effects [135]. Additional mechanistic evidence was obtained in a two-kidney, one-clip (2K1C) model of renovascular hypertension, in which eight weeks of aerobic exercise reduced SBP and renal sympathetic nerve activity, suppressed TLR4/MyD88/NF-κB signaling in the hypothalamic paraventricular nucleus, and decreased IL-1β and TNF-α expression [136]. These two studies address different components of the response to exercise: the SHR study provides evidence for exercise-associated GM remodeling, whereas the 2K1C study demonstrates modulation of TLR4/MyD88/NF-κB signaling without assessment of the GM. Therefore, they should be regarded as separate, complementary observations rather than evidence for a GM-mediated inflammatory pathway.

In a mouse model of MIRI, moderate-intensity aerobic exercise preconditioning reduced myocardial NLRP3 expression and inhibited caspase-1 and GSDMD activation, accompanied by lower IL-1β and IL-18 levels, reduced myocardial infarct size, and improved cardiac function [137]. In MI, a separate mouse study provided evidence for exercise-associated GM involvement in post-MI cardiac protection. Exercise training altered GM richness and community structure, with significant changes in taxa including *Alistipes*, *Lachnospiraceae_UCG-001*, *Ruminococcus*, *Allobaculum*, and *Oscillospiraceae UCG-005*. Exercise-associated microbial and metabolomic changes also involved *Bacteroides*, *Roseburia*, *Lachnospiraceae*, and *Ruminococcaceae*, which were correlated with microbiota-related metabolites including 3-HPA, 4-HPA, and 4-HBA. Importantly, depletion of the GM with antibiotics attenuated the cardioprotective effects of exercise, whereas fecal microbiota transplantation from exercised MI mice improved cardiac function in recipient MI mice. Further experiments showed that 3-HPA and 4-HBA supplementation improved post-MI cardiac function [138]. In patients with heart failure, exercise training was associated with reduced circulating IL-1β levels and altered inflammasome-related ASC regulation, suggesting a potential role of exercise in modulating IL-1β-associated inflammatory responses [139]. Consistent with these disease-specific findings, a recent review summarized evidence indicating that exercise may modulate NLRP3 inflammasome activation and pyroptosis, thereby reducing downstream inflammatory mediators such as IL-1β and IL-18 and potentially contributing to cardiovascular protection [128]. Taken together, these studies show that exercise-associated changes in GM composition, inflammatory markers, and cardiovascular phenotypes have been reported across different experimental and clinical settings. However, the relationships among these outcomes have not been consistently examined within the same studies.

Notably, the biological effects of IL-6 are context-dependent. Chronic and sustained elevations of circulating IL-6, as observed during persistent low-grade inflammation, are generally associated with pro-inflammatory signaling and may contribute to CVD progression. In contrast, the transient increase in IL-6 released from contracting skeletal muscle during exercise acts as a myokine and may exert metabolic and anti-inflammatory effects [140]. In this context, exercise-induced IL-6 may activate JAK2/signal transducer and activator of transcription 3 (STAT3) signaling in cardiomyocytes, with downstream regulation of Bcl-2 (heat shock protein 70) HSP70, and mitochondrial pathways contributing to reduced myocardial apoptosis and improved cardiac function [141,142]. Collectively, current studies have separately reported exercise-associated changes in GM composition, microbiota-related metabolites, and TLR4/NF-κB/NLRP3-related inflammatory signaling. However, these findings arise from different experimental systems and should be interpreted as complementary observations rather than as sequential components of an established mechanistic pathway. Direct evidence demonstrating a causal sequence from exercise-induced GM remodeling through inflammatory pathway modulation to subsequent cardiovascular benefits remains limited.

### 6.2. Exercise May Modulate IL-10- and TGF-β-Related Immunoregulatory Signaling Through the Gut Microbiota in CVD

Chronic inflammation is a key contributor to CVD progression, and maintaining the balance between pro-inflammatory and anti-inflammatory responses is essential for cardiovascular homeostasis [143]. Among these regulatory factors, IL-10 limits excessive inflammatory responses and contributes to immune homeostasis. TGF-β exerts context-dependent effects, supporting immune regulation and tissue repair while potentially promoting fibrosis and pathological cardiac remodeling when its signaling is persistent or excessive [144]. Disruption of these regulatory pathways may disturb inflammatory homeostasis and thereby contribute to the development and progression of CVD.

Regular exercise may reshape GM composition and metabolic functions, which may contribute to improved intestinal homeostasis and immune regulation. The combination of aerobic exercise and probiotic supplementation was associated with improved GM diversity, increased serum IL-10 levels, and reduced circulating LPS and IL-6 levels in obese mice [145]. SCFAs, particularly butyrate, can promote the differentiation of colonic Foxp3^+^ Treg cells and contribute to intestinal immune homeostasis [146]. Complementing these findings, Smith et al. demonstrated in mice that SCFAs regulated colonic Treg homeostasis and enhanced Treg IL-10 expression and production in a GPR43 (FFAR2)-dependent manner, supporting a link between microbial metabolites and intestinal immune regulation [147]. IL-10, a key anti-inflammatory cytokine, activates the Janus kinase (JAK)/STAT3 signaling pathway by binding to the IL-10 receptor complex, resulting in STAT3 phosphorylation (p-STAT3) and subsequent induction of suppressor of cytokine signaling 3 (SOCS3) expression [148,149]. Supporting the downstream IL-10 signaling mechanism, studies in mice have shown that *Lactobacillus rhamnosus* GG upregulates interleukin-10 receptor 2 (IL-10R2) expression, enhances IL-10 receptor-dependent STAT3 phosphorylation, and increases SOCS3 expression in the murine colon, accompanied by reduced production of pro-inflammatory cytokines such as TNF-α [150] (Figure 1). Conversely, studies in IL-10-deficient mice have shown that resident enteric bacteria are required for the development of spontaneous colitis and associated immune activation, highlighting the importance of IL-10 in restraining inflammatory responses to the intestinal microbiota [151]. Human exercise-intervention studies provide additional, although still associative, evidence linking GM changes with IL-10-related inflammatory regulation in specific cardiovascular populations. In patients with hypertension, 12 weeks of Liuzijue training increased serum IL-10 levels and altered GM composition. Correlation analysis further showed that *Bacteroides* was positively associated with IL-10, whereas *Escherichia-Shigella* was negatively associated with IL-10, suggesting that exercise-associated GM alterations may be linked to anti-inflammatory immune regulation [152]. In patients following a first MI, eight weeks of aerobic exercise training increased circulating IL-10 and improved cardiorespiratory fitness compared with usual care [153].

In addition to IL-10-mediated regulation, exercise-induced GM remodeling may influence TGF-β-related intestinal immune responses. In a microbiota-transfer study, germ-free mice colonized with microbiota from exercise-trained donors exhibited higher colonic TGF-β and Foxp3 mRNA expression and less colon shortening and mucus depletion after dextran sulfate sodium-induced colitis than recipients of sedentary-donor microbiota [111]. These findings support a contribution of exercise-altered GM to intestinal immune regulation, although they do not establish that TGF-β mediates the protective effects. Complementary evidence from a non-exercise study helps define the microbiota–TGF-β relationship. Atarashi et al. found that colonization of germ-free mice with a defined mixture of indigenous *Clostridium* strains increased active TGF-β production by colonic epithelial cells. Conditioned medium from these cells promoted Foxp3^+^ T-cell differentiation in vitro, and this effect was attenuated by TGF-β neutralization [154]. These findings provide mechanistic support for microbiota-dependent regulation of the intestinal TGF-β–Treg axis. However, the direction of exercise-associated cytokine responses appears to depend on the experimental context. Estaki et al. reported that six weeks of voluntary wheel running in healthy mice altered GM composition, increased cecal SCFA concentrations and colonic IL-10 mRNA expression, and reduced colonic TGF-β mRNA expression. Comparable microbial and immunological benefits were not observed in Muc2-deficient mice [155]. Together, these studies suggest that intestinal responses to exercise vary with host and disease context.

At the signaling level, TGF-β binding to its receptor complex induces Smad2/3 phosphorylation, followed by association with Smad4 and nuclear translocation to regulate target-gene transcription. The functional consequences of this pathway depend on the responding cell type and disease context [156]. The intestinal immunoregulatory effects observed in these experimental models should not be directly extrapolated to cardiovascular protection. Whether exercise-induced GM remodeling improves cardiovascular outcomes through TGF-β-dependent mechanisms remains to be established.

Exercise studies provide evidence of changes in TGF-β-related vascular and myocardial remodeling. In rats with T2DM, eight weeks of swimming increased aortic miR-145-5p expression and reduced TGF-β expression, accompanied by increased expression of the VSMC contractile markers SM22α and α-SMA, decreased osteopontin (OPN) expression, and reduced vascular calcification [157]. Mechanistically, Smad3-dependent induction of Smad7 in cardiac myofibroblasts provides a negative-feedback mechanism that restrains TGF-β-induced Smad2/3 activation and limits fibrotic remodeling [158]. In another study, eight weeks of moderate-intensity treadmill training attenuated myocardial fibrosis in rats with T2DM, reduced myocardial TGF-β1, p-Smad2, and p-Smad3 expression, and increased expression of the inhibitory regulator Smad7 [159]. These findings are consistent with exercise-associated modulation of VSMC phenotype and myocardial profibrotic signaling. However, neither study assessed GM, and their findings do not establish microbial mediation of these responses.

Experimental microbiota manipulation provides complementary evidence linking exercise-associated GM changes to cardiac remodeling. In mice with MI, antibiotic-mediated microbiota depletion impaired the cardioprotective effects of treadmill training, whereas transplantation of microbiota from exercised donors improved cardiac function and reduced myocardial fibrosis and collagen I expression in recipient mice [138]. These findings support a contribution of GM to exercise-related cardiac protection. Consistent with a potential link between microbiota-related metabolites and anti-inflammatory regulation, the same exercise study showed that reduced circulating TMAO was accompanied by increased myocardial IL-10 and attenuated myocardial fibrosis, whereas TMAO supplementation weakened these exercise-associated benefits [132]. This intervention provides evidence that TMAO can modify exercise-associated cardiac responses, but GM composition and TGF-β/Smad signaling were not assessed. Mechanistic evidence comes from a separate study using MI mice and primary cardiac fibroblasts without exercise intervention. TMAO exposure reduced Smurf2 expression and TGF-βRI ubiquitination in cardiac fibroblasts, accompanied by increased TGF-β RI abundance, Smad2 phosphorylation, and α-SMA and collagen I expression. Treatment with the receptor inhibitor SB431542 attenuated these responses [160]. These results support a link between TMAO and TGF-βRI/Smad2-associated fibroblast activation. Together with the exercise findings, they suggest that changes in TMAO could connect exercise to myocardial fibrotic signaling. Human evidence is comparatively limited. A four-month exercise trial in patients with chronic heart failure found no significant overall between-group differences in changes in inflammatory markers. Reductions in TGF-β, CRP, ICAM-1, and TNF-α were observed in the subgroup with idiopathic dilated cardiomyopathy [161]. These subgroup findings require cautious interpretation, and the absence of GM assessment precludes conclusions about microbial involvement.

Collectively, available evidence supports potential links between exercise-associated GM changes, microbial metabolites, and IL-10- and TGF-β-related responses. Exercise may influence these immunoregulatory and remodeling processes, while changes in GM composition and microbiota-related metabolites may accompany and potentially contribute to the cardiovascular effects of exercise. However, the GM has not yet been established as a causal mediator of exercise-induced cardiovascular protection. Intestinal immunoregulation should nevertheless be distinguished from TGF-β-associated fibrotic remodeling in cardiovascular tissues. Although animal experiments support a potential contribution of the GM to exercise-related cardiac protection, the extent to which these benefits depend on GM-mediated IL-10 or TGF-β/Smad signaling remains unresolved, particularly in humans.

## 7. Conclusions

Available evidence indicates that exercise, the GM and its related metabolites, inflammatory regulation, and cardiovascular outcomes are interconnected at multiple levels. Exercise interventions are frequently accompanied by changes in GM composition and microbial metabolic profiles, including alterations in SCFAs, BAs, and the GM-dependent TMA/TMAO pathway. These changes may occur alongside modulation of the TLR4/NF-κB/NLRP3 pro-inflammatory axis and IL-10- and TGF-β-related immunoregulatory signaling. Evidence from AS, hypertension, myocardial infarction, and MIRI further suggests that these microbial, metabolic, and inflammatory changes may be associated with cardiovascular phenotypes such as vascular inflammation, endothelial dysfunction, myocardial injury, and cardiac remodeling. Therefore, considering exercise, GM, microbial metabolites, and inflammatory regulation within an integrated research framework may provide a useful perspective for understanding the potential gut–immune–cardiovascular interactions associated with exercise.

This framework may also offer directions for future basic and clinical research. GM composition and microbiota-related metabolites may represent useful entry points for investigating the relationships among exercise adaptation, immune and inflammatory responses, and cardiovascular phenotypes. In particular, the combined assessment of GM profiles, SCFAs, TMA/TMAO, inflammatory markers, and cardiovascular functional outcomes may help identify microbial and metabolic characteristics associated with exercise responsiveness and may provide a theoretical basis for exploring more individualized exercise strategies in CVD prevention and management.

However, the current evidence primarily supports associations and potential mechanistic links rather than an established continuous causal pathway. It has not yet been demonstrated that exercise produces cardiovascular benefits through direct modulation of the GM, nor has the GM been established as a mediator between exercise and cardiovascular outcomes. This limitation is particularly relevant in humans, where studies simultaneously assessing exercise intervention, GM alterations, microbiota-related metabolites, inflammatory signaling, and cardiovascular outcomes remain limited and are often based on relatively small sample sizes. Considerable heterogeneity also exists across studies in disease characteristics, exercise modality and dose, diet, medication use, baseline fitness, geographical background, and microbiome assessment methods. Future studies should therefore include adequately powered, well-standardized randomized controlled trials and longitudinal investigations, together with metagenomic, metabolomic, transcriptomic, and other multi-omics approaches. Mediation analyses and additional mechanistic validation will also be important for clarifying the temporal relationships, biological interactions, and potential contribution of the GM to exercise-associated cardiovascular effects.

**Table 2:** Effects of Exercise Interventions on Gut Microbiota Composition and Remodeling.

Experimental Subjects	Exercise Dose	Population/Model	Intervention Method	Bacterial Community Changes	Reference
Male C57 mice	Swimming, 60 min/day, 5 days/week, 19 weeks	HFD-induced AS model	Aerobic swimming exercise with exercise-derived FMT transplantation	*Prevotellaceae_UCG-001*, *Prevotellaceae_NK3B31_group*, *Bacillus*, *Ruminococcus* ↑; *Lachnospiraceae_NK4A136_group*, *Dubosiella*, *norank_f_Erysipelotrichaceae* ↓	[162]
Carotid plaque patients/controls, *n* = 61	Adequate vs. inadequate physical activity	Human; 30 CP patients and 31 controls	Habitual physical activity assessment	Clostridiales ↑; There are differences in the characteristics related to Bacteroidetes	[163]
Male SHR	18–20 m/min, 0° incline, 60 min/day, 5 days/week, 8 weeks	8-week-old spontaneously hypertensive rats	Aerobic treadmill exercise	*Elusimicrobium* and Ruminococcaceae UCG-009 ↑; Spirochaetes and ASF356 ↓; GM composition improved.	[164]
60 adults with hypertension	10–15 × 1-min cycling at 85–90% HRR, 3 sessions/week, 12 weeks	Human; hypertension	HIIT	TMAO ↓; acetate, propionate and butyrate ↑	[102]
Patients with hypertension	12 weeks	Human; hypertension	Liuzijue exercise	F/B ratio ↓; *Bacteroides* and *Escherichia-Shigella* altered; GM dysbiosis improved	[152]
Male C57BL/6 mice	Treadmill running exercise (8 weeks)	Male C57BL/6 mice with MI induced by coronary artery ligation	Exercise training after MI; GM depletion and fecal microbiota transplantation were used for mechanistic validation	Exercise increased gut microbial richness and altered gut microbial community structure after MI; exercise-induced microbiota transplantation improved cardiac function	[138]
Male db/db mice	8 weeks, 6 days/week	8-week-old male C57BL/KS.db db/db mice with T2DM	Aerobic exercise and RE, or combined AE–RE	AE: Chao1 ↑, Proteobacteria ↓; RE: Chao1 and Shannon indices ↑, Actinobacteria↑, Helicobacter, *LachnoClostridium*, and *Anaerobium* ↓, *Roseburia* and unclassified *Ruminococcaceae* ↑; combined exercise: Shannon index ↑.	[89]
Patients with T2DM	6-month Baduanjin training	Human; 30 patients with T2DM mellitus	Baduanjin exercise intervention	*Chao1* and *Shannon indices* ↑; *Lactobacillus* and *Bifidobacterium* ↑; *Escherichia coli*, *Enterococcus*, fungi and *Candida albicans* ↓	[165]
C57BL/6 mice	17 m/min, 45 min/day, 5 days/week, 8 weeks	HFD-induced obese mice	Lacticaseibacillus paracasei N1115 combined with aerobic exercise	F/B ratio ↓; ACE, Chao1, and Shannon indices ↑; GM diversity improved	[145]
Children with obesity	8 weeks, 4 sessions/week; AE: 60–70% HRmax, 50 min/session; RT: 65–85% 1RM; combined: 20 min RT + 20 min AE/session	Human; children with obesity, aged 10–12 years	Dietary restriction combined with aerobic exercise, resistance training, or combined exercise	*Enterococcus* ↓; *Escherichia coli* ↓; *Lactobacillus* ↑; *Bifidobacterium* ↑	[94]
Sedentary lean and obese adults	6-week aerobic endurance exercise training (3–5 sessions/week, 30–60 min/session)	Human; sedentary lean and obese adults	Supervised aerobic endurance exercise	SCFA-producing bacterial genes and microbial taxa ↑; GM functional capacity improved	[95]
C57BL/6J mice	8 weeks	HFD-induced NAFLD mice	Aerobic exercise (AE) or RE	AE: F/B ratio ↓; *Escherichia-Shigella* and *Leuconostoc* decreased compared with HFD mice. RE: no reversal of HFD-associated differential genera was observed.	[125]
Male Wistar rats	10, 20, or 30 m/min, 30 min/day, 5 days/week, 8 weeks	Healthy male Wistar rats, 8–12 weeks old, 200–250 g	Low, moderate, and high-intensity aerobic exercise	Exercise intensity-dependent alterations: moderate intensity increased *Akkermansia muciniphila* and *Faecalibacterium prausnitzii*, whereas high intensity showed opposite changes.	[166]
Sedentary middle-aged and elderly women	8 weeks, 3 sessions/week, 60 min/session	Human; 30 sedentary middle-aged and elderly women, randomized into exercise and control groups (*n* = 15 each)	Flexi-Bar exercise intervention	Bacilli, Butyricicoccaceae, Ruminococcaceae, and Subdoligranulum ↑; Lachnospiraceae UCG-004 and Flavonifractor ↓; α-diversity unchanged.	[123]
CT-26 tumor-bearing mice	30 min/session, 2 sessions/day, 6 days/week, 3 weeks	Colorectal cancer transplanted tumor mouse model	swimming training	GM diversity ↑; F/B ratio ↓; butyrate-producing bacteria including *Lachnospiraceae* ↑	[167]

Note: Studies combining exercise with microbiota transplantation were included only when exercise-induced gut microbiota remodeling was specifically investigated.

## Data Availability

No new data were created or analyzed in this study.

## Abbreviations

AMI, acute myocardial infarction; Ang II, angiotensin II; AS, atherosclerosis; ASCVD, atherosclerotic cardiovascular disease; BAs, bile acids; Bcl-2, B-cell lymphoma 2; BFR, blood flow restriction; CAD, coronary artery disease; Caspase-1, cysteine-dependent aspartate-specific protease-1; COX-2, cyclooxygenase-2; CRE, cardiorespiratory exercise; CRP, C-reactive protein; CVD, cardiovascular disease; DAMPs, damage-associated molecular patterns; DBP, diastolic blood pressure; DCM, diabetic cardiomyopathy; DM, diabetes mellitus; DPP-4, dipeptidyl peptidase-4; F/B ratio, Firmicutes/Bacteroidetes ratio; FXR, farnesoid X receptor; GLP-1, glucagon-like peptide-1; GM, gut microbiota; GPR41, G protein-coupled receptor 41; GPR43, G protein-coupled receptor 43; GSDMD, gasdermin D; HDL-C, high-density lipoprotein cholesterol; HE, hematoxylin and eosin; HIIT, high-intensity interval training; HSP70, heat shock protein 70; ICAM-1, intercellular adhesion molecule-1; IFN-γ, interferon-γ; IKK, IκB kinase; IL-1β, interleukin-1β; IL-6, interleukin-6; IL-8, interleukin-8; IL-10, interleukin-10; IL-10R, interleukin-10 receptor; IL-10R2, interleukin-10 receptor 2; ImP, imidazole propionate; JAK, Janus kinase; KC, keratinocyte-derived chemokine; LDL-C, low-density lipoprotein cholesterol; LPS, lipopolysaccharide; MI, myocardial infarction; MICT, moderate-intensity continuous training; MIRI, myocardial ischemia–reperfusion injury; MyD88, myeloid differentiation primary response protein 88; NF-κB, nuclear factor kappa B; NLRP3, NOD-like receptor family pyrin domain-containing 3; NOX4, NADPH oxidase 4; OPN, osteopontin; ox-LDL, oxidized low-density lipoprotein; PAGln, phenylacetylglutamine; PINK1, PTEN-induced kinase 1; PRKN, Parkin RBR E3 ubiquitin protein ligase; ROS, reactive oxygen species; SBP, systolic blood pressure; SCFAs, short-chain fatty acids; Smad, small mothers against decapentaplegic; SOCS3, suppressor of cytokine signaling 3; STAT, signal transducer and activator of transcription; TGF-β, transforming growth factor-β; TLR4, Toll-like receptor 4; TMA, trimethylamine; TMAO, trimethylamine N-oxide; TNF-α, tumor necrosis factor-α; Treg, regulatory T cell; UC, ulcerative colitis; VCAM-1, vascular cell adhesion molecule-1; VEGF, vascular endothelial growth factor; VSMCs, vascular smooth muscle cells; ZO-1, zonula occludens-1.

## References

[1] Zhu X.Y., Shi M.Q., Jiang Z.M., Xiao L., Tian J.W., Su F.F. (2025). Global, regional, and national burden of cardiovascular diseases attributable to metabolic risks across all age groups from 1990 to 2021: An analysis of the 2021 global burden of disease study data. BMC Public Health.

[2] National Center for Cardiovascular Diseases, The Writing Committee of the Report on Cardiovascular Health and Diseases in China (2026). Summary of the China Cardiovascular Health and Disease Report 2025. China Circ. J..

[3] Li X.H., Yin F.T., Zhou X.H., Zhang A.H., Sun H., Yan G.L., Wang X.J. (2022). The Signaling Pathways and Targets of Natural Compounds from Traditional Chinese Medicine in Treating Ischemic Stroke. Molecules.

[4] Libby P. (2021). Inflammation during the life cycle of the atherosclerotic plaque. Cardiovasc. Res..

[5] Witkowski M., Weeks T.L., Hazen S.L. (2020). Gut Microbiota and Cardiovascular Disease. Circ. Res..

[6] Violi F., Cammisotto V., Bartimoccia S., Pignatelli P., Carnevale R., Nocella C. (2023). Gut-derived low-grade endotoxaemia, atherothrombosis and cardiovascular disease. Nat. Rev. Cardiol..

[7] Qi H.Y., Ma X.G., Duan R.X., Xu Y., Cui J.K. (2026). Research progress on prevention and treatment of diabetic atherosclerosis by Chinese materia medica based on NF-κB signaling pathway. Shanghai J. Tradit. Chin. Med..

[8] Xu J.X. (2024). The Role and Mechanism of Aerobic Exercise on Improving DSS-Induced Intestinal Inflammation by Regulating Mitophagy Through AMPK-SIRT1-FOXO1 Pathway. Master’s Thesis.

[9] Yang Z.Q., Qi X.Y., Qiao J. (2026). Mechanistic insights into cholesterol–bile acid metabolic dysregulation in ovarian aging. Chin. Sci. Bull..

[10] Keirns B.H., Koemel N.A., Sciarrillo C.M., Anderson K.L., Emerson S.R. (2020). Exercise and intestinal permeability: Another form of exercise-induced hormesis?. Am. J. Physiol. Gastrointest. Liver Physiol..

[11] Mailing L.J., Allen J.M., Buford T.W., Fields C.J., Woods J.A. (2019). Exercise and the Gut Microbiome: A Review of the Evidence, Potential Mechanisms, and Implications for Human Health. Exerc. Sport Sci. Rev..

[12] Longoria C.R., Guers J.J., Campbell S.C. (2022). The Interplay between Cardiovascular Disease, Exercise, and the Gut Microbiome. Rev. Cardiovasc. Med..

[13] Chen L., Wang J. (2022). Gut microbiota and inflammatory bowel disease. WIREs Mech. Dis..

[14] Liu Y., Zhao C.C., Li Z.X. (2024). Research Progress on the Prevention and Treatment of Metabolic Diseases through Improving Gut Microbiota through Exercise Training. Chin. J. Cell Biol..

[15] Tang T.W.H., Chen H.C., Chen C.Y., Yen C.Y.T., Lin C.J., Prajnamitra R.P., Chen L.L., Ruan S.C., Lin J.H., Lin P.J. (2019). Loss of Gut Microbiota Alters Immune System Composition and Cripples Postinfarction Cardiac Repair. Circulation.

[16] Koeth R.A., Wang Z., Levison B.S., Buffa J.A., Org E., Sheehy B.T., Britt E.B., Fu X., Wu Y., Li L. (2013). Intestinal microbiota metabolism of L-carnitine, a nutrient in red meat, promotes atherosclerosis. Nat. Med..

[17] Bennett B.J., de Aguiar Vallim T.Q., Wang Z., Shih D.M., Meng Y., Gregory J., Allayee H., Lee R., Graham M., Crooke R. (2013). Trimethylamine-N-oxide, a metabolite associated with atherosclerosis, exhibits complex genetic and dietary regulation. Cell Metab..

[18] Wahlström A., Sayin S.I., Marschall H.U., Bäckhed F. (2016). Intestinal Crosstalk between Bile Acids and Microbiota and Its Impact on Host Metabolism. Cell Metab..

[19] Fan Y., Pedersen O. (2021). Gut microbiota in human metabolic health and disease. Nat. Rev. Microbiol..

[20] Xie M., Li X., Lau H.C., Yu J. (2025). The gut microbiota in cancer immunity and immunotherapy. Cell. Mol. Immunol..

[21] Wang T.Y., Lan Q.S., Deng H.A., Han W.Q., Zhang R.T., Zhong J.Q. (2025). Interactions between gut microbiota and cardiovascular drugs: Effects on drug therapeutic effect and side effect. Front. Cardiovasc. Med..

[22] Guo Z., Pan J., Zhu H., Chen Z.Y. (2022). Metabolites of Gut Microbiota and Possible Implication in Development of Diabetes Mellitus. J. Agric. Food Chem..

[23] Yang Y.P., Zhang Z., Yu Y.X., Wang Y.G., Zhang Z.Q., Rong X.J., Wang H., Liu D.Y., Wang C.Y. (2026). Preliminary Investigation of an Experimental Model of Intestinal Inflammation Induced by Lipopolysaccharide in Blackrock Fish, *Sebastes schlegelii*. Prog. Fish. Sci..

[24] Guan B.Y. (2023). The Effect of Qing-Xin-Jie-Yu Granule on Atherosclerosis and Plaque Stability Based on the Gut Microbiota and Bile Acid Metabolism. Ph.D. Thesis.

[25] Ye J., Li L., Wang M., Ma Q., Tian Y., Zhang Q., Liu J., Li B., Zhang B., Liu H. (2022). Diabetes Mellitus Promotes the Development of Atherosclerosis: The Role of NLRP3. Front. Immunol..

[26] Kelly T.N., Bazzano L.A., Ajami N.J., He H., Zhao J., Petrosino J.F., Correa A., He J. (2016). Gut Microbiome Associates with Lifetime Cardiovascular Disease Risk Profile Among Bogalusa Heart Study Participants. Circ. Res..

[27] Yi C., Sun W., Ding L., Yan M., Sun C., Qiu C., Wang D., Wu L. (2022). Short-Chain Fatty Acids Weaken Ox-LDL-Induced Cell Inflammatory Injury by Inhibiting the NLRP3/Caspase-1 Pathway and Affecting Cellular Metabolism in THP-1 Cells. Molecules.

[28] Sun T., Wang Q.S., Feng J., Song H.Z. (2026). Sweet potato polysaccharides ameliorate colitis via activation of PINK1/PARK-mediated mitophagy and modulating gut microbiota. Sci. Sin..

[29] Zhang S., Zhong R., Zhou M., Li K., Lv H., Wang H., Xu Y., Liu D., Ma Q., Chen L. (2025). Mechanisms of Baicalin Alleviates Intestinal Inflammation: Role of M1 Macrophage Polarization and *Lactobacillus amylovorus*. Adv. Sci..

[30] Chen L., Gu Q.H., Jing S., Yang F., Xu Y.J., Xu Y. (2023). Effect of Jianpi Huazhi Recipe on Intestinal Microflora and Serum Inflammatory Factors in Mild-Moderate Ulcerative Colitis Patients with Pi Deficiency and Dampness-Heat Syndrome. Chin. J. Integr. Tradit. West. Med..

[31] Pepys M.B., Hirschfield G.M. (2003). C-reactive protein: A critical update. J. Clin. Investig..

[32] Munyaka P.M., Rabbi M.F., Khafipour E., Ghia J.E. (2016). Acute dextran sulfate sodium (DSS)-induced colitis promotes gut microbial dysbiosis in mice. J. Basic Microbiol..

[33] Wang X., Wu Y., Zhang X. (2026). Multidimensional Analysis of Serum Adiponectin, Leptin, and Resistin Levels and Their Correlation with Childhood Obesity Based on Gut Microbiota and Inflammatory Markers: A Single-Center Cross-Sectional Retrospective Study. Int. J. Gen. Med..

[34] Mohsenzadeh A., Pourasgar S., Mohammadi A., Nazari M., Nematollahi S., Karimi Y., Firoozbakhsh P., Mohsenzadeh H., Kamali K., Elahi R. (2025). The gut microbiota and cardiovascular disease: Exploring the role of microbial dysbiosis and metabolites in pathogenesis and therapeutics. Life Sci..

[35] Roth G.A., Mensah G.A., Johnson C.O., Addolorato G., Ammirati E., Baddour L.M., Barengo N.C., Beaton A.Z., Benjamin E.J., Benziger C.P. (2020). Global Burden of Cardiovascular Diseases and Risk Factors, 1990–2019: Update From the GBD 2019 Study. J. Am. Coll. Cardiol..

[36] Liu J.J., Liu S.L., Zhu K.Y., Ren J. (2026). Research Progress of Sinomenine in Cardiovascular Diseases. Chin. J. Integr. Med. Cardio-Cerebrovasc. Dis..

[37] Jiang S.J., Gong L., Zheng H. (2026). Research progress on the correlation between *Akkermansia muciniphila* and cardiovascular diseases. J. South. Med. Univ..

[38] Jie Z., Xia H., Zhong S.-L., Feng Q., Li S., Liang S., Zhong H., Liu Z., Gao Y., Zhao H. (2017). The gut microbiome in atherosclerotic cardiovascular disease. Nat. Commun..

[39] Du H., Xu X., Lei Q., Qian C., Wang Z., Yu X., Lei Q. (2026). Distribution of gut microbiota and prognostic value in elderly patients with coronary atherosclerosis: A preliminary culture-based study. BMC Cardiovasc. Disord..

[40] Li X., Gao H.J., Zhang L., Li P.F. (2024). Effect of combined probiotics intervention on atherosclerosis and its possible mechanism. J. Qingdao Univ..

[41] Fan J.F., You Z.H., Li J., Zhang Y.L., Liang C.H. (2026). *Pediococcus pentosaceus* ATCC33316 ameliorates atherosclerosis in ApoE^-/-^ mice by upregulating indol-3-yl pyruvic acid in the intestine. Chin. Heart J..

[42] Liang Y., Zhao L., Zhang X., Liu S., Lu P., Wang J., Chen L., Liu Y., Gao J. (2025). Lactobacillus ameliorates myocardial ischemia reperfusion injury by attenuating apoptosis, inflammation, oxidative stress, and ferroptosis. BMC Med..

[43] Cai Y. (2026). Probiotics in atherosclerosis: Mechanisms, efficacy and future directions—A review study. Front. Immunol..

[44] Koh A., Molinaro A., Ståhlman M., Khan M.T., Schmidt C., Mannerås-Holm L., Wu H., Carreras A., Jeong H., Olofsson L.E. (2018). Microbially Produced Imidazole Propionate Impairs Insulin Signaling through mTORC1. Cell.

[45] Mastrangelo A., Robles-Vera I., Mañanes D., Galán M., Femenía-Muiña M., Redondo-Urzainqui A., Barrero-Rodríguez R., Papaioannou E., Amores-Iniesta J., Devesa A. (2025). Imidazole propionate is a driver and therapeutic target in atherosclerosis. Nature.

[46] Ottosson F., Brunkwall L., Smith E., Orho-Melander M., Nilsson P.M., Fernandez C., Melander O. (2020). The gut microbiota-related metabolite phenylacetylglutamine associates with increased risk of incident coronary artery disease. J. Hypertens..

[47] Nemet I., Saha P.P., Gupta N., Zhu W., Romano K.A., Skye S.M., Cajka T., Mohan M.L., Li L., Wu Y. (2020). A Cardiovascular Disease-Linked Gut Microbial Metabolite Acts via Adrenergic Receptors. Cell.

[48] Ma H., Yang L., Liu Y., Yan R., Wang R., Zhang P., Bai Z., Liu Y., Ren Y., Li Y. (2023). Butyrate suppresses atherosclerotic inflammation by regulating macrophages and polarization via GPR43/HDAC-miRNAs axis in ApoE^-/-^ mice. PLoS ONE.

[49] Hosseinkhani F., Heinken A., Thiele I., Lindenburg P.W., Harms A.C., Hankemeier T. (2021). The contribution of gut bacterial metabolites in the human immune signaling pathway of non-communicable diseases. Gut Microbes.

[50] Liu Q.L. (2022). Macrophage Activation Induced by TMAO Through CD147/MMPs. Master’s Thesis.

[51] Delk S.C., Reddy S.T., Fogelman A.M. (2025). The intestine and cardiovascular disease. Curr. Opin. Lipidol..

[52] Du L.Y., Li Q.Y., Chen W.Z., Wang J., Fang X., Wang Y.T., Liao Z.L. (2022). Progress in Research on the Relationship between Pro-Inflammatory Gut Microbiota and Atherosclerosis. Food Sci..

[53] Miyazaki-Anzai S., Masuda M., Levi M., Keenan A.L., Miyazaki M. (2014). Dual activation of the bile acid nuclear receptor FXR and G-protein-coupled receptor TGR5 protects mice against atherosclerosis. PLoS ONE.

[54] Xue J., Allaband C., Zuffa S., Zhou D., Poulsen O., Meadows J., McDonald D., Ambre M., Ackermann G., Birmingham A. (2026). Farnesoid X receptor-dependent microbiome-bile acid signaling mediates obstructive sleep apnea-induced atherosclerosis. bioRxiv.

[55] Deussen A., Kopaliani I. (2023). Targeting inflammation in hypertension. Curr. Opin. Nephrol. Hypertens..

[56] Zhou B., He Y., Liu C.J., Li X.R. (2025). Research progress in the regulation of blood pressure by gut microbiota. Acta Microbiol. Sin..

[57] Li J., Zhao F., Wang Y., Chen J., Tao J., Tian G., Wu S., Liu W., Cui Q., Geng B. (2017). Gut microbiota dysbiosis contributes to the development of hypertension. Microbiome.

[58] Calderón-Pérez L., Gosalbes M.J., Yuste S., Valls R.M., Pedret A., Llauradó E., Jimenez-Hernandez N., Artacho A., Pla-Pagà L., Companys J. (2020). Gut metagenomic and short chain fatty acids signature in hypertension: A cross-sectional study. Sci. Rep..

[59] Pluznick J.L., Protzko R.J., Gevorgyan H., Peterlin Z., Sipos A., Han J., Brunet I., Wan L.X., Rey F., Wang T. (2013). Olfactory receptor responding to gut microbiota-derived signals plays a role in renin secretion and blood pressure regulation. Proc. Natl. Acad. Sci. USA.

[60] Natarajan N., Hori D., Flavahan S., Steppan J., Flavahan N.A., Berkowitz D.E., Pluznick J.L. (2016). Microbial short chain fatty acid metabolites lower blood pressure via endothelial G protein-coupled receptor 41. Physiol. Genom..

[61] Yin X., Duan C., Zhang L., Zhu Y., Qiu Y., Shi K., Wang S., Zhang X., Zhang H., Hao Y. (2024). Microbiota-derived acetate attenuates neuroinflammation in rostral ventrolateral medulla of spontaneously hypertensive rats. J. Neuroinflamm..

[62] Jama H.A., Rhys-Jones D., Nakai M., Yao C.K., Climie R.E., Sata Y., Anderson D., Creek D.J., Head G.A., Kaye D.M. (2023). Prebiotic intervention with HAMSAB in untreated essential hypertensive patients assessed in a phase II randomized trial. Nat. Cardiovasc. Res..

[63] Verhaar B.J.H., Wijdeveld M., Wortelboer K., Rampanelli E., Levels J.H.M., Collard D., Cammenga M., Nageswaran V., Haghikia A., Landmesser U. (2024). Effects of Oral Butyrate on Blood Pressure in Patients with Hypertension: A Randomized, Placebo-Controlled Trial. Hypertension.

[64] Wang H., Luo Q., Ding X., Chen L., Zhang Z. (2022). Trimethylamine N-oxide and its precursors in relation to blood pressure: A mendelian randomization study. Front. Cardiovasc. Med..

[65] Yu S.H., Li H., He C.L., Zhang P. (2025). Analysis of association between intestinal microbiota characteristics and risk of cardiovascular complications in hypertensive patients. Chin. J. Microecol..

[66] Tuttolomondo A., Simonetta I., Daidone M., Mogavero A., Ortello A., Pinto A. (2019). Metabolic and Vascular Effect of the Mediterranean Diet. Int. J. Mol. Sci..

[67] Huang Y.L., Li S., He Z.Y., Zhang Z.Y. (2025). Research progress on fecal microbiota transplantation for the treatment of hypertension. J. Pract. Med..

[68] Thygesen K., Alpert J.S., Jaffe A.S., Chaitman B.R., Bax J.J., Morrow D.A., White H.D. (2019). Fourth universal definition of myocardial infarction (2018). Eur. Heart J..

[69] Xia S., Liu Y., Wang M., Liu D., Zhang X., Lin L., Wen M., Ji S., Li J., Zhang X. (2025). Prognostic value of gut microbiota and low-density lipoprotein cholesterol subfractions in patients with ST-segment elevation myocardial infarction. Front. Immunol..

[70] Wang Y.L., Hao S.T., Liu Q.H., Li Y.M., Yang X.H., Xia Y., Yuan X.H. (2025). Mechanisms of gut microbiota dysbiosis in exacerbating myocardial infarction via T cell immunity and inflammation and the effects of probiotic intervention. Chin. J. Extracorpor. Circ..

[71] Su H.M., Li J.X., Wu J.F., Wu C., Sheng S.H., Wang R.S., Guo Z.Q., Shen S.C., Gao Y.F., Tian B.D. (2026). Morchella polysaccharide ameliorates myocardial infarction inflammation in mice through increasing gut microbiota-derived 12-HEPE. Acta Pharmacol. Sin..

[72] Organ C.L., Li Z., Sharp T.E., Polhemus D.J., Gupta N., Goodchild T.T., Tang W.H.W., Hazen S.L., Lefer D.J. (2020). Nonlethal Inhibition of Gut Microbial Trimethylamine N-oxide Production Improves Cardiac Function and Remodeling in a Murine Model of Heart Failure. J. Am. Heart Assoc..

[73] Li X., Sun Y., Zhang X., Wang J. (2019). Reductions in gut microbiota-derived metabolite trimethylamine N-oxide in the circulation may ameliorate myocardial infarction-induced heart failure in rats, possibly by inhibiting interleukin-8 secretion. Mol. Med. Rep..

[74] Chen L., Wang F., Zhang H., Qin S., Sui M., Ishigami T. (2025). Lactulose for prevention of cardiovascular events after myocardial infarction: A retrospective cohort study. JRSM Cardiovasc. Dis..

[75] Dong Z.H., Xuan X.Y., Feng L.M. (2025). Research progress on the regulation of signaling pathways related to myocardial ischemia-reperfusion injury by monomer of traditional Chinese medicine. China J. Tradit. Chin. Med. Pharm..

[76] Zhao J., Zhang Q., Cheng W., Dai Q., Wei Z., Guo M., Chen F., Qiao S., Hu J., Wang J. (2023). Heart-gut microbiota communication determines the severity of cardiac injury after myocardial ischaemia/reperfusion. Cardiovasc. Res..

[77] Ñahui Palomino R.A., Vanpouille C., Costantini P.E., Margolis L. (2021). Microbiota-host communications: Bacterial extracellular vesicles as a common language. PLoS Pathog..

[78] Wang J., Hu K., Lu H., Chen K., Zhang J., Wu S., Kang L., Xie J., Xu B. (2026). Invasion of gut-derived escherichia coli extracellular vesicles exacerbates myocardial ischemia/reperfusion injury. Gut Microbes.

[79] Zhang J., Liu C., Wang Y., Chen Y., Shang H., Zheng W., Jin L., Xie P., Li Y., Liu Y. (2026). Bacteroides acidifaciens exacerbates cardiac ischemia/reperfusion injury via the microbial-host isozyme DPP4. Cell Host Microbe.

[80] Du H., Liu X., Shen J., Yuan H., Zhang H., Xi G., Li Y., Wang Y., Zhang J., Yang C. (2025). Flavonifractor Plautii or Its Metabolite Desaminotyrosine as Prophylactic Agents for Alleviating Myocardial Ischemia/Reperfusion Injury. Adv. Sci..

[81] Wang Y.J., Zhou J.D., Su Q.Y., Yao J.C., Su R.Q., Qin G.F., Zhang G.M., Liang H.B., Feng S., Zhang J.C. (2025). Effect and mechanism of Xintong Granules in ameliorating myocardial ischemia-reperfusion injury in rats by regulating gut microbiota. Zhongguo Zhong Yao Za Zhi.

[82] Danilo C.A., Constantopoulos E., McKee L.A., Chen H., Regan J.A., Lipovka Y., Lahtinen S., Stenman L.K., Nguyen T.V., Doyle K.P. (2017). *Bifidobacterium animalis* subsp. *lactis* 420 mitigates the pathological impact of myocardial infarction in the mouse. Benef. Microbes.

[83] Wang J., Zhang H., Yuan H., Chen S., Yu Y., Zhang X., Gao Z., Du H., Li W., Wang Y. (2024). Prophylactic Supplementation with *Lactobacillus reuteri* or Its Metabolite GABA Protects Against Acute Ischemic Cardiac Injury. Adv. Sci..

[84] Clauss M., Gérard P., Mosca A., Leclerc M. (2021). Interplay Between Exercise and Gut Microbiome in the Context of Human Health and Performance. Front. Nutr..

[85] Woods J.A., Vieira V.J., Keylock K.T. (2009). Exercise, inflammation, and innate immunity. Immunol. Allergy Clin. N. Am..

[86] Qian X., Guo Y., Sun P. (2025). Mechanisms of exercise in preventing cardiovascular diseases: Insights from gut microbiota characteristics in pathological states of cardiovascular diseases. Rev. Endocr. Metab. Disord..

[87] Long H. (2024). Study on the Effect and Mechanism of Aerobic Exercise in Alleviating Symptoms of Inflammatory Bowel Disease Model Mice. Master’s Thesis.

[88] Kasimu A., Huang Y., Wang D., Jiang Y., Liu Y., Song Y., Zhang Y., Chen X., Li B. (2026). Exercise pretreatment intervention alleviates intestinal dysbiosis and improves intestinal dysfunction in stroke rats. BMC Gastroenterol..

[89] Zhang Q. (2021). Effect of Different Exercise Modulates Microbiota on Inflammatory Response in Type 2 Diabetic Mice. Master’s Thesis.

[90] Chen J., Guo Y., Gui Y., Xu D. (2018). Physical exercise, gut, gut microbiota, and atherosclerotic cardiovascular diseases. Lipids Health Dis..

[91] Liu Z., Liu H.Y., Zhou H., Zhan Q., Lai W., Zeng Q., Ren H., Xu D. (2017). Moderate-Intensity Exercise Affects Gut Microbiome Composition and Influences Cardiac Function in Myocardial Infarction Mice. Front. Microbiol..

[92] Schönke M., Ying Z., Kovynev A., In Het Panhuis W., Binnendijk A., van der Poel S., Pronk A.C.M., Streefland T.C.M., Hoekstra M., Kooijman S. (2023). Time to run: Late rather than early exercise training in mice remodels the gut microbiome and reduces atherosclerosis development. FASEB J..

[93] Ramos C., Hunter K., Walton G.E., Whitham A., Camp N., Poveda C., Gibson G.R., Hough J., Magistro D. (2026). Associations Between the Gut Microbiota and Physical Activity, Sedentary Behaviour and Physical Function in Community-Dwelling Older Adults. J. Aging Res..

[94] Wang P., Liu B.L., Hu Z.Y., Liu Y., Jiang Q., Zhang P. (2024). Effects of dietary restriction combined with different exercises on inflammatory factors and gut microbiota in obese children. Chin. J. Sch. Health.

[95] Allen J.M., Mailing L.J., Niemiro G.M., Moore R., Cook M.D., White B.A., Holscher H.D., Woods J.A. (2018). Exercise Alters Gut Microbiota Composition and Function in Lean and Obese Humans. Med. Sci. Sports Exerc..

[96] Motiani K.K., Collado M.C., Eskelinen J.J., Virtanen K.A., Löyttyniemi E., Salminen S., Nuutila P., Kalliokoski K.K., Hannukainen J.C. (2020). Exercise Training Modulates Gut Microbiota Profile and Improves Endotoxemia. Med. Sci. Sports Exerc..

[97] Mazur-Kurach P., Frączek B., Klimek A.T. (2022). Does Multi-Strain Probiotic Supplementation Impact the Effort Capacity of Competitive Road Cyclists?. Int. J. Environ. Res. Public Health.

[98] Duah O.B., Young L., de La Serre C., Zhu H., Xu H. (2026). Functional Capacity and Gut Microbiota Shifts in Heart Failure Patients Following Cardiac Rehabilitation. J. Clin. Med..

[99] Gao C., Yue Y., Wu D., Zhang J., Zhu S. (2025). Effects of high-intensity interval training versus moderate-intensity continuous training on cardiorespiratory and exercise capacity in patients with coronary artery disease: A systematic review and meta-analysis. PLoS ONE.

[100] Jalma M.D., Sianipar I.R., Goenarjo R.A., Paramita N., Santoso D.I.S. (2026). HIIT versus MICT on Blood Pressure and Cardiac Biomarkers in L-NAME-induced Hypertensive Rats. Int. J. Exerc. Sci..

[101] Yudiansyah A.G., Tanjaya K., Wendry J., Anggarkusuma M.V., Wulandari K.S., Adrianta M., Nugroho M.B. (2026). Cardioprotective and anti-inflammatory properties of blood flow restriction training in older or high-risk cardiovascular disease population: Systematic review and meta-analysis. J. Sports Med. Phys. Fit..

[102] Ghardashi Afousi A., Asvadi Fard M., Sabouri A. (2026). High-intensity interval training is associated with improvements in ambulatory blood pressure, arterial stiffness, and gut-derived metabolites in adults with hypertension. J. Hypertens..

[103] Liu Z., Li J., Liu H., Tang Y., Zhan Q., Lai W., Ao L., Meng X., Ren H., Xu D. (2019). The intestinal microbiota associated with cardiac valve calcification differs from that of coronary artery disease. Atherosclerosis.

[104] Yu Y.X., Wang Q.Y., Yuan Y.T., Luo Y.X., Zhou C.Y., Zhang Y.J., Huang W. (2026). Mechanism of electroacupuncture in improving lipid metabolism in diabetic cardiomyopathy mice via activation of the intestinal FXR/FGF15 pathway. J. Li-Shizhen Tradit. Chin. Med..

[105] Lin J.N., Lin Z.F., Liu Q.L., Pan Q.S., Ma S.Q., Zhong K.Q., Zeng R.F., Xi X.T., Li J., Chen R. (2025). Effects of Yiqi Huoxue Jiedu Fang on macrophage phenotype in patients with platinum-resistant ovarian cancer through regulating IL-6. J. Beijing Univ. Tradit. Chin. Med..

[106] Meng R., Feng W., Li Z.J., Chen Y., Wu C.X., Wang X.P., Li J.A. (2026). The Mechanism of Jin-Ju Tea in Ameliorating Type 2 Diabetes in Rats through Gut Microbiota. Sci. Technol. Food Ind..

[107] Wu R., Zu W., Wei L., Wu R., Su S., A R., A T., E N., Li H., Hu R. (2025). Metagenomics and metabolomics integrated to explore the protective mechanisms of Mongolian medicine Zadi-5 in myocardial ischemic model rats. Front. Microbiol..

[108] Cho J.M., Park S.K., Satheesh Babu A.K., Mookherjee S., Hansen M., Petersen C., Zhong Y., Jalili T., Robeson M.S., Wankhade U.D. (2026). Late-in-life treadmill training mitigates gut microbiome imbalances and cardiovascular disease risk in mice. Am. J. Physiol. Gastrointest. Liver Physiol..

[109] Yang T., Santisteban M.M., Rodriguez V., Li E., Ahmari N., Carvajal J.M., Zadeh M., Gong M., Qi Y., Zubcevic J. (2015). Gut Dysbiosis Is Linked to Hypertension. Hypertension.

[110] Cook M.D., Martin S.A., Williams C., Whitlock K., Wallig M.A., Pence B.D., Woods J.A. (2013). Forced treadmill exercise training exacerbates inflammation and causes mortality while voluntary wheel training is protective in a mouse model of colitis. Brain Behav. Immun..

[111] Allen J.M., Mailing L.J., Cohrs J., Salmonson C., Fryer J.D., Nehra V., Hale V.L., Kashyap P., White B.A., Woods J.A. (2018). Exercise training-induced modification of the gut microbiota persists after microbiota colonization and attenuates the response to chemically-induced colitis in gnotobiotic mice. Gut Microbes.

[112] Liu J., Yu H., Jan Y.K. (2026). A systematic review and meta-analysis of the mechanism of action of Tai Chi on cardiovascular disease: Evidence map of aerobic and mind-body exercise pathways. Sci. Rep..

[113] Tang W.H., Kitai T., Hazen S.L. (2017). Gut Microbiota in Cardiovascular Health and Disease. Circ. Res..

[114] Escobar C., Aldeguer X., Vivas D., Manzano Fernández S., Gonzalez Caballero E., Garcia Martín A., Barrios V., Freixa-Pamias R. (2025). The gut microbiota and its role in the development of cardiovascular disease. Expert. Rev. Cardiovasc. Ther..

[115] Lewis C.V., Taylor W.R. (2020). Intestinal barrier dysfunction as a therapeutic target for cardiovascular disease. Am. J. Physiol. Heart Circ. Physiol..

[116] Dmytriv T.R., Storey K.B., Lushchak V.I. (2024). Intestinal barrier permeability: The influence of gut microbiota, nutrition, and exercise. Front. Physiol..

[117] Fountoulakis P.N., Theofilis P., Vlachakis P.K., Karakasis P., Pamporis K., Sagris M., Dimitroglou Y., Tsioufis P., Oikonomou E., Tsioufis K. (2025). Gut Microbiota in Heart Failure-The Role of Inflammation. Biomedicines.

[118] Mukherjee T.K., Malik P., Singh R., Haribhai Patel V., Kumar R. (2026). Nordihydroguaiaretic acid attenuates TNFα-dependent intercellular adhesion molecule-1 expression in cultured human umbilical vein endothelial cells via targeting TNFα-PI3K-NF-κB-ICAM1 pathway. Avicenna J. Phytomed..

[119] Emdad L., Das S.K., Dasgupta S., Hu B., Sarkar D., Fisher P.B. (2013). AEG-1/MTDH/LYRIC: Signaling pathways, downstream genes, interacting proteins, and regulation of tumor angiogenesis. Adv. Cancer Res..

[120] Wu W., Wang H., Jiao G., Yue J., Wang G. (2017). Aerobic Exercise Suppresses Atherosclerosis Through Adiponectin-Nuclear Transcription Factor κB Pathway in Apolipoprotein E-deficient Mice. Am. J. Med. Sci..

[121] Huang W.-C., Tung C.-L., Yang Y.-C.S.H., Lin I.H., Ng X.E., Tung Y.-T. (2022). Endurance exercise ameliorates Western diet–induced atherosclerosis through modulation of microbiota and its metabolites. Sci. Rep..

[122] Jiang L., Liu P., Wang M., Deng Q., Wang J., Jiang Y., Zhu Y., Meng H., Lu X., Kong X. (2024). Effect of high-intensity intermittent rehabilitation training on physical function, gut microbiome and metabolite after percutaneous coronary intervention in patients with coronary heart disease. Front. Cardiovasc. Med..

[123] Mai Y.N. (2022). Effects of 8-week Flexi-Bar Exercise Intervention on Vascular Function and Gut Microbiota in Sedentary Middle-Aged and Elderly Women. Master’s Thesis.

[124] Gianaros P.J., Wan L., DeCataldo M.K., Hidalgo C.M., Scudder M.R., Grove G., Shell A., Kang C.R., Wu-Chung E.L., Marsland A.L. (2026). Effects of a year-long aerobic exercise intervention on neuroendocrine, autonomic, and neural correlates of stress, emotion, and cardiovascular disease risk in midlife adults. J. Sport Health Sci..

[125] Wu F.J., Lei S.L., Li X.H., Yang Y. (2026). Aerobic and resistance exercise interventions in a mouse model of nonalcoholic fatty liver disease: Correlation between gut microbiota and irisin. Chin. J. Tissue Eng. Res..

[126] Liu Y.P., Fan Y. (2026). Research progress on the role of NLRP3 inflammasome in atherosclerosis. J. Med. Theory Pract..

[127] Swanson K.V., Deng M., Ting J.P. (2019). The NLRP3 inflammasome: Molecular activation and regulation to therapeutics. Nat. Rev. Immunol..

[128] Li X., Weng R.Q., Zhao J.L., Gu X.D., Liu S.D. (2025). Research progress of NLRP3 inflammasome in cardiovascular diseases. J. Med. Res..

[129] Yuan Z., Li H., Ruan B.X., Huang H., Li Y., Wang J. (2026). Macrophage-derived galectin-3 contributes to pyroptosis, apoptosis and necroptosis through TLR4/MyD88/NF-κB/NLRP3 during atherosclerosis. Clin. Transl. Med..

[130] Kim K.W., Ivanov S., Williams J.W. (2020). Monocyte Recruitment, Specification, and Function in Atherosclerosis. Cells.

[131] Chen M.L., Zhu X.H., Ran L., Lang H.D., Yi L., Mi M.T. (2017). Trimethylamine-N-Oxide Induces Vascular Inflammation by Activating the NLRP3 Inflammasome Through the SIRT3-SOD2-mtROS Signaling Pathway. J. Am. Heart Assoc..

[132] Zhang H., Meng J., Yu H. (2017). Trimethylamine N-oxide Supplementation Abolishes the Cardioprotective Effects of Voluntary Exercise in Mice Fed a Western Diet. Front. Physiol..

[133] Zhang Y., Wang G., Li R., Liu R., Yu Z., Zhang Z., Wan Z. (2023). Trimethylamine N-oxide aggravated cognitive impairment from APP/PS1 mice and protective roles of voluntary exercise. Neurochem. Int..

[134] Zhang Y., Wang A.C., Wang D.G. (2024). Research progress on activation mechanism of NLRP3 inflammasome and its role in cardiovascular disease. J. Qiqihar Med. Univ..

[135] Xia W.J., Xu M.L., Yu X.J., Du M.M., Li X.H., Yang T., Li L., Li Y., Kang K.B., Su Q. (2021). Antihypertensive effects of exercise involve reshaping of gut microbiota and improvement of gut-brain axis in spontaneously hypertensive rat. Gut Microbes.

[136] Qi J., Yu X.J., Fu L.Y., Liu K.L., Gao T.T., Tu J.W., Kang K.B., Shi X.L., Li H.B., Li Y. (2019). Exercise Training Attenuates Hypertension Through TLR4/MyD88/NF-κB Signaling in the Hypothalamic Paraventricular Nucleus. Front. Neurosci..

[137] Wang Y., Li Y., Chen C., Zhang H., Liu W., Wu C., Chen H., Li R., Wang J., Shi Y. (2024). Moderate-intensity aerobic exercise inhibits cell pyroptosis to improve myocardial ischemia-reperfusion injury. Mol. Biol. Rep..

[138] Zhou Q., Deng J., Pan X., Meng D., Zhu Y., Bai Y., Shi C., Duan Y., Wang T., Li X. (2022). Gut microbiome mediates the protective effects of exercise after myocardial infarction. Microbiome.

[139] Butts B., Butler J., Dunbar S.B., Corwin E., Gary R.A. (2018). Effects of Exercise on ASC Methylation and IL-1 Cytokines in Heart Failure. Med. Sci. Sports Exerc..

[140] Pedersen B.K., Febbraio M.A. (2008). Muscle as an endocrine organ: Focus on muscle-derived interleukin-6. Physiol. Rev..

[141] Zhang Q.Q., Chen F.H., Wang F., Di X.M., Li W., Zhang H. (2022). A Novel Modulator of the Renin-Angiotensin System, Benzoylaconitine, Attenuates Hypertension by Targeting ACE/ACE2 in Enhancing Vasodilation and Alleviating Vascular Inflammation. Front. Pharmacol..

[142] Chen J., Jia S., Xue X., Guo C., Dong K. (2024). Gut microbiota: A novel target for exercise-mediated regulation of NLRP3 inflammasome activation. Front. Microbiol..

[143] Camarda N.D., Ibarrola J., Biwer L.A., Jaffe I.Z. (2024). Mineralocorticoid Receptors in Vascular Smooth Muscle: Blood Pressure and Beyond. Hypertension.

[144] Frangogiannis N.G. (2022). Transforming growth factor-β in myocardial disease. Nat. Rev. Cardiol..

[145] Zhao H.Y., Cao S.J., Qi J.N., Bian Y.Q., Zhang J.Q., Gao Y.Q., Feng Z.Y., Sun W.J., Xue Y.L., Wang Y.H. (2025). Ameliorative Effect of Lacticaseibacillus paracasei Combined with Aerobic Exercise on Obesity in Mice. Food Sci..

[146] Furusawa Y., Obata Y., Fukuda S., Endo T.A., Nakato G., Takahashi D., Nakanishi Y., Uetake C., Kato K., Kato T. (2013). Commensal microbe-derived butyrate induces the differentiation of colonic regulatory T cells. Nature.

[147] Smith P.M., Howitt M.R., Panikov N., Michaud M., Gallini C.A., Bohlooly Y.M., Glickman J.N., Garrett W.S. (2013). The microbial metabolites, short-chain fatty acids, regulate colonic Treg cell homeostasis. Science.

[148] Carlini V., Noonan D.M., Abdalalem E., Goletti D., Sansone C., Calabrone L., Albini A. (2023). The multifaceted nature of IL-10: Regulation, role in immunological homeostasis and its relevance to cancer, COVID-19 and post-COVID conditions. Front. Immunol..

[149] Solouki S., Gorgani-Firuzjaee S., Jafary H., Delfan M. (2024). Efficacy of high-intensity interval and continuous endurance trainings on cecal microbiota metabolites and inflammatory factors in diabetic rats induced by high-fat diet. PLoS ONE.

[150] Mirpuri J., Sotnikov I., Myers L., Denning T.L., Yarovinsky F., Parkos C.A., Denning P.W., Louis N.A. (2012). *Lactobacillus rhamnosus* (LGG) regulates IL-10 signaling in the developing murine colon through upregulation of the IL-10R2 receptor subunit. PLoS ONE.

[151] Sellon R.K., Tonkonogy S., Schultz M., Dieleman L.A., Grenther W., Balish E., Rennick D.M., Sartor R.B. (1998). Resident enteric bacteria are necessary for development of spontaneous colitis and immune system activation in interleukin-10-deficient mice. Infect. Immun..

[152] Wu S., Zheng C., Liu N., Deng T., Wang J., Qi L., Xia L. (2023). Liuzijue training improves hypertension and modulates gut microbiota profile. Front. Cardiovasc. Med..

[153] Ribeiro F., Alves A.J., Teixeira M., Miranda F., Azevedo C., Duarte J.A., Oliveira J. (2012). Exercise training increases interleukin-10 after an acute myocardial infarction: A randomised clinical trial. Int. J. Sports Med..

[154] Atarashi K., Tanoue T., Shima T., Imaoka A., Kuwahara T., Momose Y., Cheng G., Yamasaki S., Saito T., Ohba Y. (2011). Induction of colonic regulatory T cells by indigenous Clostridium species. Science.

[155] Estaki M., Morck D.W., Ghosh S., Quin C., Pither J., Barnett J.A., Gill S.K., Gibson D.L. (2020). Physical Activity Shapes the Intestinal Microbiome and Immunity of Healthy Mice but Has No Protective Effects against Colitis in MUC2(-/-) Mice. mSystems.

[156] Humeres C., Venugopal H., Frangogiannis N.G. (2022). Smad-dependent pathways in the infarcted and failing heart. Curr. Opin. Pharmacol..

[157] Xu G.G., Ma C.L., Yang Y. (2024). Involvement of microRNA-145/TGF-β in the regulation of swimming exercise on vascular calcification in type 2 diabetic rats. Sheng Li Xue Bao.

[158] Humeres C., Shinde A.V., Hanna A., Alex L., Hernández S.C., Li R., Chen B., Conway S.J., Frangogiannis N.G. (2022). Smad7 effects on TGF-β and ErbB2 restrain myofibroblast activation and protect from postinfarction heart failure. J. Clin. Investig..

[159] Wang S.Q., Li D., Yuan Y. (2019). Long-term moderate intensity exercise alleviates myocardial fibrosis in type 2 diabetic rats via inhibitions of oxidative stress and TGF-β1/Smad pathway. J. Physiol. Sci..

[160] Yang W., Zhang S., Zhu J., Jiang H., Jia D., Ou T., Qi Z., Zou Y., Qian J., Sun A. (2019). Gut microbe-derived metabolite trimethylamine N-oxide accelerates fibroblast-myofibroblast differentiation and induces cardiac fibrosis. J. Mol. Cell. Cardiol..

[161] Byrkjeland R., Nilsson B.B., Westheim A.S., Arnesen H., Seljeflot I. (2011). Inflammatory markers as related to disease severity in patients with chronic heart failure: Limited effects of exercise training. Scand. J. Clin. Lab. Investig..

[162] Men J., Li H., Cui C., Ma X., Liu P., Yu Z., Gong X., Yao Y., Ren J., Zhao C. (2025). Fecal bacteria transplantation replicates aerobic exercise to reshape the gut microbiota in mice to inhibit high-fat diet-induced atherosclerosis. PLoS ONE.

[163] Ouyang W., Tang B., He Y., Wu H., Yang P., Yin L., Li X., Li Y., Huang X. (2024). Mediation effect of gut microbiota on the relationship between physical activity and carotid plaque. Front. Microbiol..

[164] Liang Q., Zhang Y., Chen Y., Chen J.Y., Xue P.W., Jiang B., Zhang L.L., Li J., Zhong D.L., Jin R.J. (2025). Effects of aerobic treadmill exercise on gut microbiota in spontaneously hypertensive rats. Chin. J. Hypertens..

[165] Zhang R.H., Liu S.W., Gao J.J. (2024). The role of Baduanjin training in improving type 2 diabetes based on gut microbiota, serum inflammatory factors, and intestinal mucosal barrier function. Chin. J. Microecol..

[166] Sylviana N., Karisa P., Romadona N.F., Megantara I. (2025). Optimising exercise intensity for gut health: Effect on microbiota composition, barrier integrity and inflammation in male Wistar rats. Exp. Physiol..

[167] Li J.P., Xie Q.R., Wen Y., Chen H.C., Peng J., Shen A.L. (2022). Effect of Swimming on Gut Microbiota and Inflammation in Colorectal Cancer Cells Transplanted Mice. Rehabil. Med..

